# Photodynamic therapy promotes hypoxia‐activated nitrogen mustard drug release

**DOI:** 10.1002/smo.20240010

**Published:** 2024-06-11

**Authors:** Ran Wang, Maomao He, Zongwei Zhang, Tian Qiu, Yue Xi, Xiaolong Zeng, Jiangli Fan, Wen Sun, Xiaojun Peng

**Affiliations:** ^1^ State Key Laboratory of Fine Chemicals Frontiers Science Center for Smart Materials‐Oriented Chemical Engineering Dalian University of Technology Dalian China; ^2^ Max Planck Institute for Polymer Research Mainz Germany; ^3^ Ningbo Institute of Dalian University of Technology Ningbo China

**Keywords:** cascade reaction, chemotherapy, hypoxia‐activated, photodynamic therapy, prodrug

## Abstract

Photodynamic therapy (PDT) has become a promising method for tumor treatment due to its non‐invasive and high spatiotemporal selectivity. However, PDT is still hindered by reactive oxygen species deficiency, because solid tumors feature a hypoxic microenvironment. PDT combined with hypoxia‐activated chemotherapy drugs can effectively induce tumor death, overcoming the limitations of the sole PDT for the fight against hypoxia. Herein, we designed a nanosystem (PCe6AZOM) that enhances the release of hypoxia‐activated drugs (AZOM) by PDT. Under hypoxic conditions, the azo bond of AZOM is cleaved by azo reductase, releasing highly cytotoxic AZOM and resulting in a significant increase in intratumor drug concentration. Meanwhile, the commercial photosensitizer Ce6 can aggravate the oxygen‐poor state during the PDT process and further cause more AZOM release. Moreover, the cascade reactions in the nanosystem could activate singlet oxygen and enhance drug release through 660 nm light laser irradiation, contributing to more effective induction of tumor apoptosis and tumor growth retardation in vitro and in vivo.

## Graphical abstract

We have designed a nanosystem (PCe6AZOM) that can enhance the release of hypoxic‐activating drugs (AZOM) through PDT. Under the irradiation of 660 nm irradiation, the photosensitizer Ce6 can produce reactive oxygen species, aggravate the hypoxia level in the tumor, and promote the release of hypoxia prodrug AZOM, which shows excellent anti‐tumor effects in vivo and in vitro.

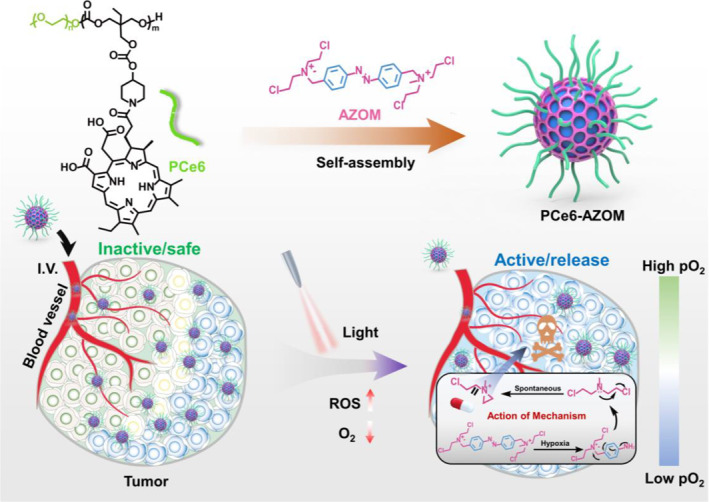

## 1 INTRODUCTION

Photodynamic therapy (PDT) is a useful therapeutic approach for cancer because of its safety, spatiotemporal selectivity, and noninvasiveness. The principle of PDT is to inhibit tumor growth through cytotoxic reactive oxygen species (ROS) produced by the reaction of photosensitizers (PSs) with surrounding O_2_ under light. Unfortunately, tumors are highly aggressive and relapsing.[^1^, ^2^, ^3^, ^4^] In addition, the abnormally rapid proliferation of tumor cells and dysfunctional new angiogenesis cause an imbalance between rapid oxygen consumption and insufficient oxygen supply, resulting in lower oxygen concentrations within the solid tumor.[^5^, ^6^] To solve the above problems, considerable studies have mainly focused on modifying PSs that could improve tumor treatment efficiency by increasing the yield of ROS.^[7]^ However, simply improving the performance of PSs is still not enough for practical applications. Furthermore, the PDT process rapidly consumes O_2_ in the tumor, causing microvascular collapse, greatly reducing the ability of O_2_ to transport into the tumor, and further exacerbating hypoxia.^[8]^ Therefore, further exploration of novel therapeutic strategies is still needed to alleviate the hypoxia limitation of PDT.

Chemotherapy, as the most common method of clinical tumor treatment, can be combined with PDT to enhance the treatment efficiency of PDT under hypoxia.[^9^, ^10^] However, paclitaxel (PTX) and camptothecin (CPT) et al. are commonly used chemotherapeutic drugs that usually lack tumor targeting and can cause side effects on normal cells and tissues. The development and application of stimulus‐responsive prodrugs improve the targeting of tumors and realize the precise treatment of tumors by chemotherapeutic drugs. Prodrugs in response to a variety of stimuli, generally fall into two broad categories, including external stimuli (e.g., light, sound) or internal stimuli of tumor‐specific microenvironments (e.g., low pH, overexpressed enzymes).[^11^, ^12^, ^13^, ^14^, ^15^] Especially, hypoxic‐responsive prodrugs as effective antitumor drugs are on the rise, providing an opportunity to solve the problem of low efficiency of PDT treatment caused by tumor hypoxia.[^16^, ^17^, ^18^, ^19^] As azo reductase‐specific cracking groups, azo benzene derivatives can recognize hypoxia environments and have been used as the connecting arm of hypoxia response prodrugs and probes for hypoxia recognition.[^18^, ^20^, ^21^] Azo benzene derivatives can be selectively reduced by overexpressing azo reductase in the hypoxic environment of tumors, releasing chemotherapeutic drugs with active status and pharmacological cytotoxicity, to improve the ability of tumor targeting. Therefore, the PDT‐aggravated tumor hypoxia by consuming O_2_ in the tumor may further promote the release of hypoxia‐activated prodrug through a cascade reaction, which is beneficial to achieve a more effective therapeutic effect for practical application.

**SCHEME 1:**
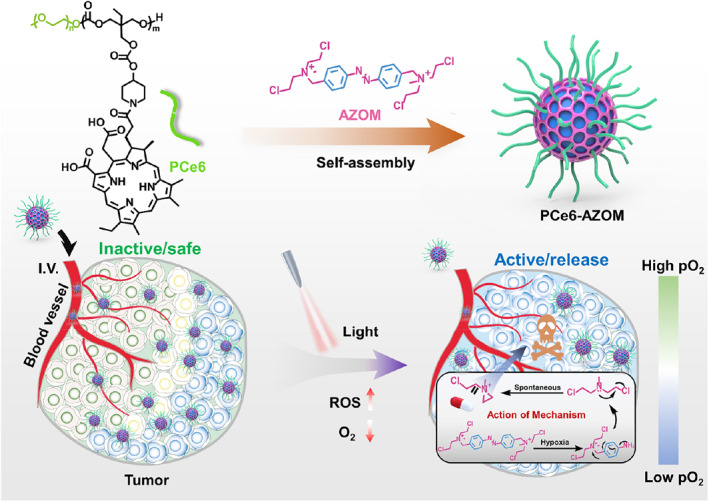
Schematic illustration of the self‐assembly of PCe6AZOM, and the activation of the AZOM prodrug in PDT‐induced hypoxia in tumors for potentiated chemo‐photodynamic combined therapy.

In this work, we constructed a multifunctional nanosystem (called PCe6AZOM), for synergistic cancer therapy with PDT‐amplified hypoxic‐activated chemotherapy. The nanosystem contains chlorine e6 (Ce6)‐modified amphiphilic polymer and azobenzene‐linked chemotherapeutic drugs (AZOM) (Scheme 1). Ce6 is connected to theamphiphilic polymer (MPEG‐b‐PTMCP) by amide bonds, and AZOM is wrapped in the hydrophobic cavity of the nanoparticles through electrostatic interactions and hydrophobic interactions in supramolecular assembly. Singlet oxygen (^1^O_2_) generated by encapsulated Ce6 causes tumor apoptosis under 660 nm light irradiation. Furthermore, PDT process greatly exacerbates the intratumor hypoxia microenvironment, amplifying AZOM release by chemotherapy drugs, which induces DNA damage and combines with PDT to enhance the synergistic antitumor effect.

## 2 RESULTS AND DISCUSSION

### 2.1 Preparation and characterization of PCe6 and PCe6AZOM

To validate our design, we first prepared the diblock copolymer MPEG‐b‐PTMCP. In brief, trimethylpropane imidazolide carbonate (TMPIC) was first synthesized from trimethylol propane and N,N′‐carbonyldiimidazole, and then transesterified with 4‐hydroxypiperidin‐1‐tert‐butyl carboxylate to form piperidine additional cyclic carbonate monomer (TMCP‐Boc). Block copolymer (MPEG‐b‐PTMCP‐Boc) and diblock copolymer MPEG‐b‐PTMCP were synthesized by ring‐opening polymerization (ROP) and deprotection, respectively (Figure S1). Then, the Ce6‐modified amphiphilic polymer PCe6 was successfully synthesized using an amidation reaction of MPEG‐b‐PTMCP with the commercial PS Ce6. Subsequently, a hypoxia‐responsive prodrug (AZOM) with azo as a hypoxic reaction linker was prepared through a four‐step reaction process (Figure S2). PCe6, AZOM, and all the intermediates were characterized by NMR (Figures S3‐S9).

**FIGURE 1:**
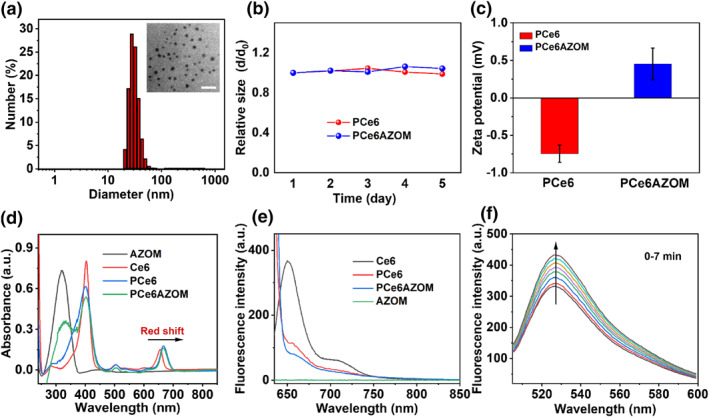
(a) Dynamic light scattering size distribution of PCe6AZOM nanoparticles. Transmission electron microscopy images of PCe6AZOM nanoparticles. Scale bar: 20 nm. (b) Size changes of PCe6 and PCe6AZOM in PBS solution with prolonged time. (c) The zeta potential of PCe6 and PCe6AZOM nanoparticles. (d) UV‐vis spectra of AZOM, free Ce6, PCe6, and PCe6AZOM nanoparticles. (e) Fluorescence spectra of AZOM, free Ce6, PCe6, and PCe6AZOM nanoparticles. (f) The production of singlet oxygen by PCe6AZOM nanoparticles in PBS determined by fluorescence enhancement of singlet oxygen sensor green.

PCe6AZOM nanoparticles containing AZOM were prepared by electrostatic interaction between the quaternary amine cation of AZOM and the carboxyl group of Ce6 wrapped in the hydrophobic cavity of the polymer. Dynamic light scattering (DLS) showed that the average hydrodynamic diameter of PCe6AZOM and PCe6 nanoparticles was 31.49 nm and 20.41 (Figure 1a and Figure S11a), respectively. PCe6AZOM and PCe6 nanoparticles were both spherical‐like structures with homogeneous size distributions verified by transmission electron microscopy (TEM) (Figure 1a and Figure S11b). In addition, the size of PCe6AZOM and PCe6 nanoparticles remained almost unchanged within 5 days (Figure 1b), indicating the good colloidal stability of the prepared nanoparticles. Suitable particle size, zeta potential (Figure 1c), and good stability are conducive to the long‐term blood circulation of nanoparticles in vivo and enhance tumor accumulation through the enhanced permeability and retention (EPR) effect, which is conducive to achieving better tumor accumulation, and more effective tumor treatment efficiency. The prodrug AZOM can be activated and 1, the 6‐elimination reaction occurs under hypoxic conditions, resulting in the formation of highly toxic nitrogen mustard (Figure S10).

PCe6AZOM showed an absorption peak similar to AZOM at 330 nm, and there were two characteristic absorption bands similar to that of free Ce6 at 400 and 660 nm (Figure 1d). While PCe6 nanoparticles only demonstrated the characteristic absorption peak of Ce6, indicating the payload of AZOM in PCe6AZOM. The slight red‐shift of nanoparticle absorption and the decrease in fluorescence were due to the slight aggregation of Ce6 in the nanoparticles. The absorption peak near 660 nm was located in the “therapeutic window” (650–900 nm), giving the nanoparticles the same therapeutic potential as free Ce6.^[22]^ The emission wavelength of PCe6AZOM extended to the near‐infrared region of 700 nm (Figure 1e), which can be used for monitoring the nanoparticle distribution. Subsequently, singlet oxygen sensor green (SOSG) was used to verify that PCe6AZOM nanoparticles inherited the ^1^O_2_ generation capacity from free Ce6. Under the laser irradiation of 660 nm (30 mW/cm^2^), the fluorescence of the mixed solution of SOSG and PCe6AZOM at 528 nm gradually increased with the light laser irradiation time (0–7 min) (Figure 1f), indicating that PCe6AZOM could effectively produce ^1^O_2_ under the laser irradiation.

### 2.2 Cellular uptake assay

**FIGURE 2:**
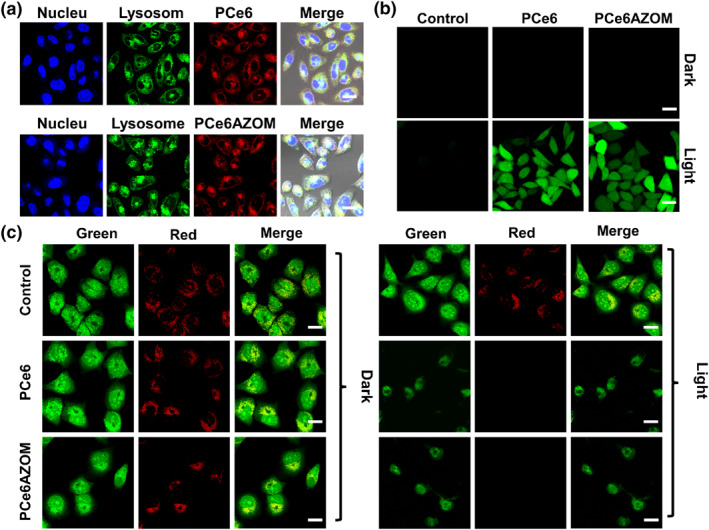
(a) Intracellular distribution of PCe6 or PCe6AZOM nanoparticles (red fluorescence) at a concentration of 20 μg/mL. Lysosomes were stained with commercial lysosome tracker dye (green fluorescence). Nuclei were stained with DAPI (blue fluorescence). (b) MCF‐7 cells stained with DCFH‐DA dye after the treatment of PBS, PCe6 or PCe6AZOM nanoparticles (20 μg/mL). (c) Images of lysosomal integrity after the treatment of PBS, PCe6 or PCe6AZOM nanoparticles (20 μg/mL). Green fluorescence of acridine orange monomer represents intact lysosomes and red fluorescence of AO dimer represents damaged lysosomes. Red‐light laser irradiation (660 nm, 30 mW/cm^2^, 5 min) was conducted after different treatments. Scale bar: 20 μm.

The performance of PCe6AZOM in cancer treatment was further investigated due to its excellent physical properties. The entry of PCe6AZOM into MCF‐7 cells was first studied using confocal laser scanning microscopy (CLSM) (Figure S12). An obvious red fluorescence signal can be observed in MCF‐7 cells at 1–4 h, indicating that PCe6AZOM was continuously internalized by cells over time. Flow cytometry showed that the maximum fluorescence intensity at 8 h (Figure S13), and remained at 12 h, which demonstrated that the nanoparticles had a long retention time and could continuously monitor the cell state. In addition, PCe6 and PCe6AZOM nanoparticles can be well localized in the lysosomes attributed to the endocytosis of cells (Figure 2a). Lysosome localization is conducive to cancer cell death caused by irreversible oxidative damage to organelles caused by the PDT process.

To further verify the ability of nanoparticles to produce ROS in cells, we selected dichlorodihydrofluorescein (DCFH) as the intracellular ROS probe. DCFH enters the cell to form 2,7‐dichlorofluorescein diacetate (DCFH‐DA). When ROS is present, it is quickly oxidized to dichlorofluorescein (DCF), which emits bright green fluorescence under light. No green fluorescence was detected in all groups in the dark, indicating that ROS was not generated in the tumor cells. It is noteworthy that PCe6 and PCe6AZOM showed obvious green fluorescence after 5 min laser irradiation at 660 nm (Figure 2b and Figure S14A), while no fluorescence signal was observed in the control group. These results indicated that PCe6 and PCe6AZOM nanoparticles effectively induced ROS production in cells under laser irradiation. Furthermore, to verify that ROS produced by nanoparticles caused irreversible oxidative damage to the lysosomes, the integrity of the lysosomes was evaluated using acridine orange (AO) (Figure 2c and Figure S14B). After PBS, PCe6 or PCe6AZOM nanoparticles incubated MFC‐7 cells for 4 h, the red fluorescence of AO in CLSM images indicated that the lysosomes were not damaged. The absence of red fluorescence and nuclear contraction in AO indicated that the lysosome damage was caused by ROS produced by PCe6AZOM nanoparticles after laser irradiation.

### 2.3 In Vitro cytotoxicity of PCe6AZOM

**FIGURE 3:**
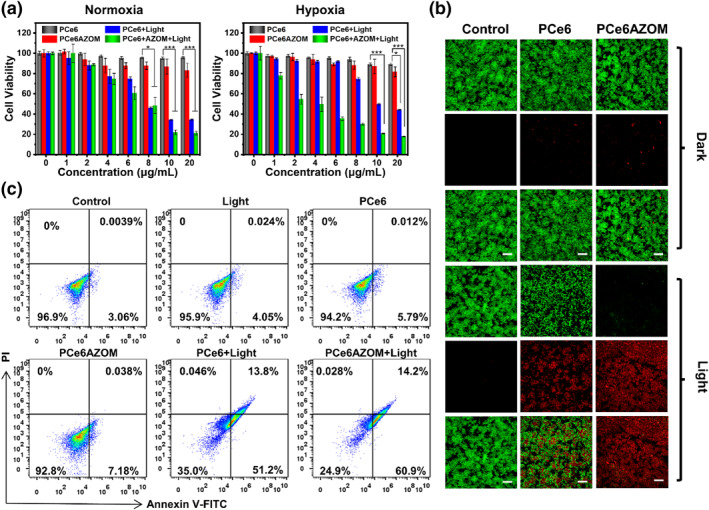
(a) Cell viability treated with PCe6 and PCe6AZOM. Light laser irradiation (660 nm, 30 mW/cm^2^, 5 min). (b) Live (green)/dead (red) staining after incubation with PBS, PCe6, and PCe6AZOM (20 μg/mL). Scale bar: 200 μm. (c) Flow cytometry analysis apoptosis of MCF‐7 cells. Light was conducted after cells were incubated with PCe6 and PCe6AZOM (20 μg/mL).

The PDT‐mediated lysosome damage in living cells would cause the release of lysosomal enzymes and ROS in the cytoplasm, leading to cell apoptosis and death. Thus, we used a cytotoxicity experiment to explore the effect of PCe6AZOM. The results showed that the cell activity of MCF‐7 was above 80% at the concentration of PCe6 and PCe6AZOM reached 20 μM under normal oxygen (Figure 3a). Therefore, the two nanoparticles had low toxicity in dark. In contrast, after exposure to red light (660 nm, 30 mW/cm^2^, 5 min), cytotoxicity increased with increasing nanoparticle concentration, and survival rate was only 17.8% at 20 μM, which could be attributed to intracellular ROS production and chemotherapeutic drugs activation. Notably, PCe6AZOM exhibited higher cytotoxicity than PCe6‐treated cells under hypoxia due to the activation of AZOM. In addition, the PDT process further exacerbated the lack of oxygen, promoting the release of more AZOM. These results indicated that cascade‐responsive chemo‐photodynamic therapy could significantly improve the inhibition of tumor cell growth. Subsequently, CLSM imaging was used to evaluate the cell activity after different treatments. PBS, PCe6, and PCe6AZOM cultured cells showed bright green fluorescence and negligible red fluorescence, indicating that they were almost living cells. After light laser irradiation, PCe6AZOM showed a greater proportion of red fluorescence and a lower proportion of green fluorescence than PCe6‐treated MCF‐7 cells (Figure 3b and Figure S15), suggesting that the increased proportion of cancer cell death caused by DNA damage from AZOM produced the combined effect of tumor inhibition, owing to rapid oxygen consumption and enhanced hypoxia environment during PDT. The levels of the intracellular DNA damage marker *γ*‐H2AX were evaluated after different treatments by immunofluorescence staining. Enhanced intracellular green fluorescence indicated increased levels of *γ*‐H2AX in DNA damage caused by PCe6AZOM with light (Figure S16).

The remarkable anticancer effect of PCe6AZOM encouraged us to investigate the way that induced cancer cell death. We monitored the apoptosis‐inducing ability of PCe6AZOM by flow cytometry. Almost no apoptotic signal was produced in cells treated with PBS, PCe6, and PCe6AZOM in the dark and light (660 nm, 30 mW/cm^2^, 5 min), suggesting that light did not affect cells (Figure 3c). However, the cells treated with PCe6 and PCe6AZOM under light showed obvious apoptosis, with the proportion of dying being 65.0% and 76.1%, respectively. Similar results were observed from confocal imaging (Figure S17). The higher apoptosis ratio of PCe6AZOM than PCe6 was attributed to apoptosis caused by the activation of AZOM, which exerted cytotoxic activity.

### 2.4 In Vivo biodistribution studies

**FIGURE 4:**
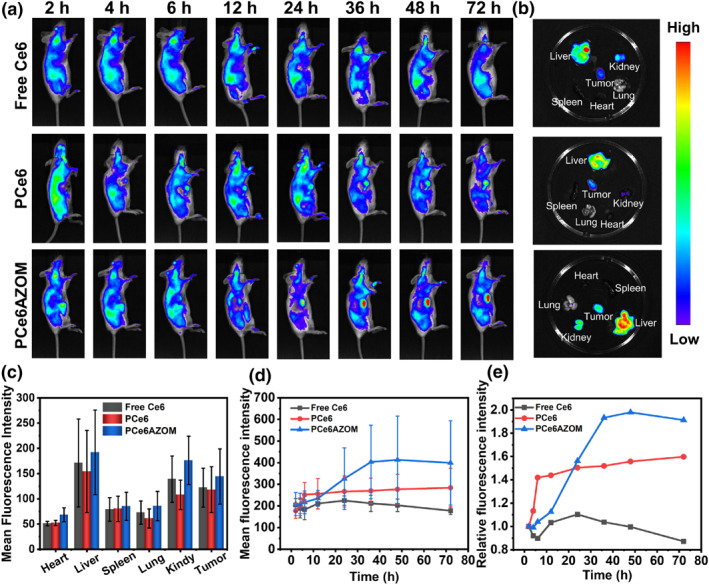
(a) *F*luorescence imaging of free Ce6, PCe6, and PCe6AZOM as a function of time. (b) Biodistribution and (c) fluorescence intensity of mice organs (d) Average fluorescence intensity of tumor after the mice were intravenously injected with free Ce6, PCe6 and PCe6AZOM. (e) Relative fluorescence intensity of free Ce6, PCe6, and PCe6AZOM in tumor.

The excellent performance of PCe6AZOM encouraged us to further explore its application in vivo. Firstly, we injected PCe6AZOM intravenously into a tumor‐bearing mouse to study its biological distribution. Tumor site showed fluorescence signa at 6 h and the fluorescence intensity was the strongest at 48 h (Figure 4a), indicating that PCe6AZOM could accumulate at the tumor site. The metabolism of nanoparticles was the reason why the fluorescence signal was weakened at 72 h. Furthermore, the tumor and normal tissues were imaging in vitro (Figure 4b). Fluorescence signals were mainly distributed in the tumor, liver, and kidney. This was also demonstrated by quantizing the average fluorescence signals in each tissue (Figure 4c). Thus, the nanoparticles could accumulate at tumor sites and be metabolized by the liver and kidney. In contrast to free Ce6 and PCe6, which demonstrated poor targeting ability for tumors, PCe6AZOM showed a higher signal‐to‐noise ratio at tumor site (Figure 4e). The good targeting was attributed to the EPR effect of PCe6AZOM nanoparticles, which is conducive to improving treatment efficiency and decreasing side effects.

### 2.5 In Vivo antitumor activity of PCe6AZOM

**FIGURE 5:**
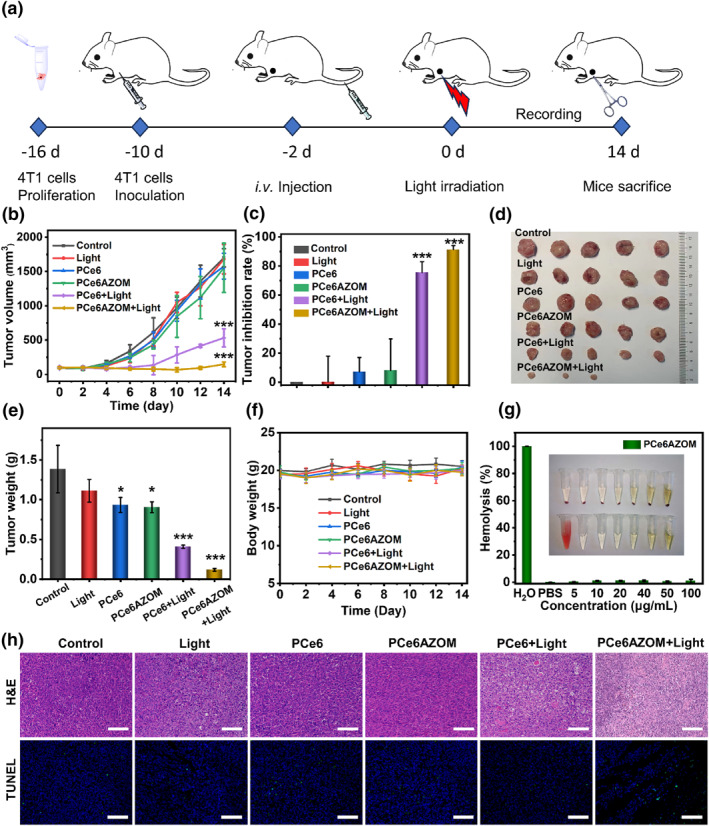
(a) 4T1 tumor volume changes during 14 days of treatment. (b) Tumor inhibition rate tumors from all groups. (c) Photographs of 4T1 tumor from all groups. (d) Changes in tumor weight after treatments. (e) Body weight changes in each group. Data are presented as mean values ± SD (*n* = 5). (f) Hemolysis of PCe6AZOM at different concentrations. (g) Representative H&E staining and immunohistochemistry staining of transferase dUTP Nick end labeling. Scale bar: 100 μm. Each group had 5 mice (*n* = 5).

The 4T1 tumor murine model was constructed and randomly divided into 6 groups: Control, Light, PCe6, PCe6AZOM, PCe6 with Light, PCe6AZOM with Light to evaluate the antitumor effect of PCe6AZOM. Similar to the control group and the light group, the tumor volume of mice injected with PCe6 and PCe6AZOM nanoparticles increased significantly within 14 days. The PCe6 with Light group showed only moderate anticancer ability (Figure 5a). Excitingly, compared with other groups, PCe6AZOM nanoparticles showed the most significant inhibitory effect with a tumor inhibition rate 91.24% (Figure 5b), which clearly indicated that PDT and chemotherapy combination was significantly superior to sole PDT. Among the in vitro tumors of each group, the PCe6AZOM laser irradiation group had the smallest tumor volume and weight, and even completely ablated the tumors of two tumor‐bearing mice (Figure 5c,d). Histological examination was also performed to evaluate the therapeutic effect of PCe6AZOM. In tumor tissues stained with hematoxylin and eosin (H&E), a large area of nuclei‐free tumor cells with obvious death could be seen after light treatment with PCe6AZOM (Figure 5g). In addition, the percentage of apoptosis induced by deoxynucleotidyl transferase dUTP nick end labeling (TUNEL) staining was significantly higher thanother groups. These results confirmed that the PDT effect of PCe6AZOM induced hypoxia under light, which triggered AZOM activation and induced DNA alkylation to inhibit tumor cell proliferation, thereby achieving the promising antitumor effect.

In addition, no significant weight abnormalities were observed in the mice during treatment (Figure 5e). No hemolysis was observed in PCe6AZOM, indicating the good stability in the blood of organisms, which greatly reduced the side effects caused by off‐target. No obvious pathological abnormality was found in normal organs. To further demonstrate the biosafety of the nanosystem, we performed hematologic analyses and routine blood analysis. The biochemical blood indexes of PCe6AZOM and other groups were within the normal range (Figure S18, S19), which confirmed that PCe6AZOM had lower systemic toxicity and side effects. Histological analysis showed no pathological differences and significant tissue damage in the images of each treatment group (Figure S20), demonstrating the high biocompatibility of PCe6AZOM in mice within the range of experimental doses.

## 3 CONCLUSION

In summary, we developed a smart PDT‐induced hypoxic cascade activation chemotherapy platform to enhance tumor phototherapy efficacy. Particularly, an azo‐linked nitrogen mustard prodrug was first constructed and then self‐assembled into Ce6‐modified polymers in an aqueous solution. This polymer‐based nanosystem can effectively produce ROS to aggravate tumor hypoxia and promote drug release. The biocompatible nanosystem exhibited the significant efficiency of inhibting tumor growth in vitro *and* in vivo. The tumor inhibition rate of PDT combined with chemotherapy was up to 91.24%. This ingenious strategy provides design guidelines for programmed cascade stimulus‐responsive drug delivery systems, which could result in excellent anticancer effects in combination with other therapeutic methods in the future.

## CONFLICT OF INTEREST STATEMENT

The authors declare no conflicts of interest.

## ETHICS STATEMENT

All protocols for animal studies conformed to the Guide for the Care and Use of Laboratory Animals and approved by the Dalian University of Technology Animal Care and Use Committee (DUT2020‐028).

## Supporting information

Supporting Information S1

## Data Availability

The data that support the findings of this study are available in the supplementary material of this article.
