# Supplementary material for: Photodynamic therapy promotes hypoxia‐activated nitrogen mustard drug release
Source: Smart Mol. 2024 Jun 11;2(3):e20240010. doi: 10.1002/smo.20240010 (PMC12118211; doi:10.1002/smo.20240010)
Supplement: Supplementary file 1 — Supporting Information S1 [file SMO2-2-e20240010-s001.docx]

Supporting Information

Photodynamic Therapy Promotes Hypoxia-activated Nitrogen Mustard Drugs Release

*Ran Wang^+^, Maomao He^+^, Zongwei Zhang, Tian Qiu, Yue Xi, Xiaolong Zeng, Jiangli Fan, Wen Sun*, Xiaojun Peng*

1. **Materials and Instruments**

Most of the regents and chemicals used in synthesis and all the other solvents used in this study were of analytical grade purchased from Energy Chemical Co. , (3-(4,5-dimethyl-2-thiazolyl)-2,5-diphenyl-2-H-tetrazolium bromide (MTT) 1,3-diphenylisobenzofuran (DPBF) were purchasedfrom Energy Chemical Co. Chlorin e6 (Ce6), Acridine Orange (AO) were purchased from MACKLIN. 2, 7-dichlorofluorescein diacetate (DCFH-DA) Detection Kit, Calcein AM/propidium iodide (PI) Detection Kit, Annexin V-FITC/PI apoptosis detection kit, 4’, 2-(4-Amidinophenyl)-6-indolecarbamidine dihydrochloride (DAPI), Lyso-Tracker Green were purchased from Beyotime Biotechnology Co. (China). Dulbecco’s modified Eagle’s medium (DMEM) and penicillin-streptomycin solution and fetal bovine serum (FBS) were purchased from Gibco (shanghai, China). All tumor cells used for experiments were purchased from Institute of Basic Medical Sciences (IBMS) of the Chinese Academy of Medical Sciences, including murine mammary carcinoma cells (4T1), human breast cancer cells (MCF-7) and human hepatoma cells (HepG-2).

H^1^-NMR spectra of all chemical intermediates and last molecule were performed by Bruker Avance III 400 spectrometer. Absorption and emission spectra of the samples were detected with a Lambda 35 UV-visible spectrophotometer (PerkinElmer) and a VAEIAN CARY Eclipse fluorescence spectrophotometer (Serial No. FL0812-M018), respectively. The structure of nanoparticles images were performed on a HT7700 EXALENS transmission electron microscopy (TEM) and the diameter was determined by dynamic light scattering (DLS) on a Zetasizer Nano ZS90. Light irradiation was performed by 660 nm LED laser device (specific model: GYH-BT-22W) and light densitometer (specific model: CEL-FZ-A) was used detected light power. Confocal laser scanning microscope (CLSM) images were obtained by Olympus FV3000. The fluorescence images of animal were captured on a NightOWL II LB983 living imaging system.

1. **Synthesis of PCe6**

**Figure S1.** Synthetic route of PCe6.

**2.1 Synthesis of PCe6**

**Synthesis of TMP carbonate imidazole carboxylate (TMPIC)**

Trimethylolpropane (TMP) (5 g, 37 mmol) was dissolved in 100 mL acetone by stirring at room temperature for 10 min. The flask was flowed by constant N2, using a

long needle, and equipped with a powder funnel on top of the needle. Then, 1,1’-carbonyldiimidazole (CDI) (14 g, 86 mmol) was slowly added over a period of 1 h at room temperature with high stirring. The next portion was added after the complete dissolution of the previous addition. After that, a white cloudy solution was obtained with a further stirring at room temperature for 1 h. The stirring was stopped and the mixture was keep for 1 h. White precipitate formed, which was collected by filtration, washed with diethyl ether and dried under vacuum to afford a fluffy white powder (5.46 g, 58%).

**Synthesis of propargyl-TMP-carbonate (TMCP-Boc)**

TMPIC (1.00 g, 5.74 mmol), N-Boc-4-piperidinol (1.73 g, 8.61 mmol) and CsF (18 mg, 0.11 mmol) were added into a round-bottom flask with flame and argon purge. Subsequently, 25 mL acetone was added under argon and further vortexed for 24 h at 20 °C. The insoluble substance in the reaction system was removed by filtration. The filtrate was collected, concentrated and subjected to column chromatography (dichloromethane/ethyl acetate = 2/1) to obtain a colorless liquid, which was further purified by crystallization from n-hexane at -20 °C to give clean monomer as a white crystal (1.48 g, 67 %).

**Synthesis of methoxy polyethyleneglycol-polycarbonate (MPEG-b-PTMCP-Boc)**

In a nitrogen-filled glovebox, a 50 ml round-bottom flask equipped with a magneticstir bar was charged with MPEG (0.601 g, 0.12 mmol), TMCP-Boc (1.16 g, 3.0 mmol), and TU (55 mg, 0.15 mmol). 6 ml THF (0.5 M) was then added and the reaction mixture was stirred until all of the chemicals completely dissolved. Next, DBU (15 µL, 0.10 mmol) was added and the reaction mixture was further stirred at 30℃ for 24 h. Afterwards, the mixture was precipitated in diethyl ether and the solid was collected following centrifugation and decanting the supernatant. This process was repeated twice to obtain the desired product as a white solid (1.44 g, 82 %).

**Deprotection of MPEG-b-PTMCP-Boc**

MPEG-b-PTMCP-Boc (0.5 g, 0.05 mmol) was dissolved in mixed solvent of 5 mL dichloromethane (DCM) and 5 mL TFA at -10 °C under Ar protection. The solution was further stirred at 25 °C for 1 h and concentrated by vacuum rotary evaporation. The product was redissolved in 5 mL deionized water, then the solution was dialyzed (MW cutoff, 1000 Da) in deionized water for 48 h and lyophilized to afford a white powder (0.38 g, 91%).

**Figure S2.** Synthetic route of pro-drug AZOM.

**2.2 Synthesis of pro-drug AZOM**

**Synthesis of Compound 1**

4-Nitrobenzyl alcohol (6.0 g, 39 mmol) was added to NaOH (70 mL, 5.7 M) aqueous solution, and Zn power (5.0 g, 75 mmol) were added slowly and the mixture was heated to reflux for 10 h with stirring. The solid were separated by filtration under reduced pressure and then suspended in hot methanol until the dissolving completely. After cooled to room temperature, the **compound 1** as bright orange solid were obtained by filtration, yield 4.1 g, 43.4%. ^1^H NMR (400 MHz, MeOD) δ 7.88 (s, 4H), 7.54 (s, 4H), 4.69 (s, 4H), 3.30 (dd, J = 3.1, 1.6 Hz, 4H).

**Synthesis of** **Compound 2**

**Compound 1** (2.0 g, 8.3 mmol) was dissolved in anhydrous THF (20 mL) and cooled with an ice-water bath under nitrogen. Triphenylphosphine (PPh_3_) (3.15 g, 43.8 mmol) and N-bromosuccinimide (NBS) (2.49 g, 43.8 mmol) were added to the solution with vigorously stirring, respectively. The solvent evaporated on a rotary evaporator then the residue was purified by column chromatography with silica gel to yield **Compound 2** as a orange solid. Yield 2.3 g, 75.7%. ^1^H NMR (400 MHz, CDCl_3_) δ 7.89 (d, J = 8.4 Hz, 4H), 7.54 (d, J = 8.4 Hz, 4H), 4.56 (s, 4H).

**Synthesis of Compound 3**

**Compound 2** (1 g, 2.7 mmol) and 2,2'-(methylazanediyl)diethanol (971.3 mg, 8.2 mmol) in 10 mL anhydrous acetonitrile was stirred in dark at room temperature for overnight. After filtration, the **Compound 3** was obtained as a white solid,.Yield 1.2 g, 72.6%. ^1^H NMR (400 MHz, DMSO-d_6_) δ 8.01 (d, J = 8.4 Hz, 4H), 7.88 (d, J = 8.4 Hz, 4H), 5.37 (s, 4H), 4.80 (s, 4H), 3.58 (dt, J = 13.3, 5.0 Hz, 4H), 3.42 (dt, J = 13.6, 5.0 Hz, 4H), 3.07 (s, 6H).

**Synthesis of** **Compound 4**

Thionyl chloride (10 mL) was slowly added dropwise to a solution of **Compound 3** (254 mg, 0.5 mmol) in CH_2_Cl_2_ (20 mL) with an ice-water bath. The reaction mixture was stirred at room temperature for 3 days. After solvent evaporation under reduced pressure, the residue was recrystallized from CH_3_OH/Et_2_O several times to obtain **Compound 4** as a orange solid. Yield, 240 mg, 71.4%. ^1^H NMR (400 MHz, DMSO-d_6_) δ 8.03 (s, 4H), 7.85 (s, 4H), 4.85 (s, 4H), 4.22 (s, 8H), 3.89 (s, 4H), 3.75 (s, 4H), 3.15 (s, 6H).

**3. Sample preparation**

**Preparation of PCe6 and** **PCe6AZOM Nanoparticles**

PCe6AZOM nanoparticles were prepared through adding THF (200 μL included 1 mg AZOM) dropwise to an ultrapure water solution (2 mL). Then the mixed solution was stirred in the dark for 24 h. Then the mixed solution was dialyzed against water for 48 h to remove organic solvents (MW cutoff of the dialysis bag, 3500 Da). The PCe6 nanoparticles were carried out as the above methods except for adding AZOM.

**4. Singlet Oxygen Detection *in vitro***

The singlet oxygen generated by the PCe6AZOM was detected using Singlet Oxygen Sensor Green reagent (SOSG). The mixed solution of PCe6AZOM and SOSG was irradiated with 660 nm light irradiation with a density of 30 mW/cm^2^ for various time, and fluorescence spectra were recorded every 1 min by fluorescence spectrophotometer immediately.

1. **Cell experiments**

**5.1 Cell culture**

MCF-7, 4T1 and HepG-2 were cultured in DMEM supplemented with 10% FBS and supplemented with 1% antibiotics (streptomycin/penicillin). All the cells were maintained in a humidified atmosphere of 5% CO_2_ in air at 37 °C. Hypoxia environment were implemented with hypoxia-mimicking CoCl_2_ according to the lecture.

**5.2 Cell co-location Imaging**

4T1 cells were cultured with DAPI and Lysosome Green (a commercial lysosome fluorescent dye) in the dark for 15 min. Then, 4T1 cells were incubated with PCe6 and PCe6AZOM in the dark for 8 h. Confocal laser scanning microscope (CLSM) was used to show the distribution of PCe6 and PCe6AZOM in cells. The excitation wavelength of DAPI is 405 nm and emission wavelength is 420-480 nm. The excitation wavelength of Lysosome Green is 488 nm and emission wavelength is 500-550 nm. The excitation wavelength of PCe6 and PCe6AZOM is 640 nm and emission wavelength is 660-750 nm.

**5.3 Intracellular ROS Level Detection**

The MCF-7 cells were seeded into confocal dishes at the density of 1×10^4^ cells per dish then cultured for 24 h. The old medium was replaced with a fresh medium containing PBS, PCe6, PCe6AZOM (20 μM) for another 4 h. The dishes were washed thrice with PBS and replaced with fresh media containing DCFH after with or without irradiation (660 nm, 30 mW/cm^2^, 5 min). All samples were imaged to give the level of intracellular ^1^O_2_ by using CLSM.

**5.4 Lysosomes disruption assay**

Acridine orange (AO) was used for detection the lysosomes integrity of the MCF-7 cells after different treatments. The 1×10^4^ cells were seeded into per confocal dishes then cultured for 24 h. The cells were incubated with different treatments including the following: Control, cells were incubated with PBS; Light, cells were irradiated with 660 nm red light (30 mW/cm^2^, 5 min); PCe6, cells were incubated with 20 μM PCe6 at 37 ºC for 8 h; PCe6AZOM, cells were incubated with 20 μM PCe6AZOM at 37 ºC for 8 h; PCe6+Light, cells were incubated with 20 μM PCe6 at 37 ºC for 4 h and then further incubated for another 4 h after irradiated with 660 nm red light (30 mW/cm^2^, 5 min); PCe6AZOM+Light, cells were incubated with 20 μM PCe6AZOM at 37 ºC for 4 h and then further incubated for another 4 h after irradiated with 660 nm red light (30 mW/cm^2^, 5 min). The cells of different groups were washed with PBS trice and then stained with AO for 20 min. All samples were imaged to give the integrity of lysosomes by using CLSM.

**5.5 Living/dead staining**

MCF-7 cells were seeded into 35 mm confocal dishes (1×10^5^ cells per dish) and cultured with DMED at 37 ℃ for 24 h. After washed with PBS thrice, the old medium was replaced with fresh DMED (2 mL) containing PBS , PCe6 (20 g/mL) or PCe6AZOM (20 μg/mL) incubated for 4 h. The light groups were exposed with 660 nm irradiation at a density of 30 mW/cm^2^ for 5 min. The cells further incubated 4 h after irradiation, the cells were stained with AM and PI for 20 min and the viability of tumors was observed by using CLSM. The excitation wavelength of AM is 488 nm and emission wavelength is 505-545 nm. The excitation wavelength of PI is 488 nm and emission wavelength is 600-700 nm.

**5.6 Apoptosis Detected by Flow** **Cytometer and CLSM**

MCF-7 cells were seeded into 6-well plates with a density of 1×10^5^ cells per well and further cultured for 24 h. The cells were incubated with different treatments including the following: Control, cells were incubated with PBS; Light, cells were irradiated with 660 nm red light (30 mW/cm^2^, 5 min); PCe6, cells were incubated with 20 μM PCe6 at 37 ºC for 8 h; PCe6AZOM, cells were incubated with 20 μM PCe6AZOM at 37 ºC for 8 h; PCe6+Light, cells were incubated with 20 μM PCe6 at 37 ºC for 4 h and then further incubated for another 4 h after irradiated with 660 nm red light (30 mW/cm^2^, 5 min); PCe6AZOM+Light, cells were incubated with 20 μM PCe6AZOM at 37 ºC for 4 h and then further incubated for another 4 h after irradiated with 660 nm red light (30 mW/cm^2^, 5 min). Then the cells were trypsinized, washed in new medium and centrifuged at 1500 RPM for 5 min. The cells were resuspended in 1 mL binding buffer containing Annexin V-FITC and PI. After treatment, the fluorescence intensity of cells was detected by flow cytometry to evaluate apoptosis effect.

In the Annexin V-FITC/PI fluorescence imaging study, the cells were seed into 35 mm confocal dishes at a density of 1×10^5^ cells per dish. Other procedures and grouping are the same as flow cytometry except that cells trypsinized are not required. And fluorescence imaging is performed directly by CLSM after staining with Annexin V-FITC/PI for 20 min. Annexin VFITC was excited with a 488 nm laser, detected in the range from 500 to 540 nm; PI was excited with a 488 nm laser, detected in the range from 650 to 690 nm.

**5.7 *In Vitro* Cytotoxicity experiment**

The cytotoxicity *in vitro* was measured by using the MTT assay. MCF-7 cells, 4T1 cells and HepG-2 cells were plated at 1×10^4^ cells per well in a 96-well plate, respectively, and then incubated for 24 h. The cells were treated with PCe6 or PCE6AZOM at the concentration from 0 to 20 μM in 100 μL DMEM to replace the old medium under nomoxia or hypoxia. The hypoxia environment was mimicked by using 100 μL DMEM medium containing 100 μM cobalt chloride (CoCl_2_). After incubation for 4 h, the cells were exposed to 660 nm LED red light (30 mW/cm^2^, 5 min). Further incubated for 1 h and removed medium out, MTT (0.5 mg/mL in 100 μL DMEM) reagent was added for 4 h at 37 °C, and DMSO (100 μL) was added to each well to dissolve the precipitated formazan violet crystals at 37 °C. After that, the absorpation of 490 was measured by a Bio-Rad microplate reader and the cell viability was evaluated by the following equation to evaluate:

Cell viability (%) = ($\frac{{OD}_{ps}-{OD}_{blank control}}{{OD}_{control}-{OD}_{blank control}}$) × 100%

**6. *In vivo* experiments**

**6.1 Blood analysis experiments**

After intravenous injection of different reagents for 48 h, each mice of different groups were sacrificed and the blood collected for toward blood biochemistry assay and complete blood routine analysis. The following groups: (1) Control, the mice were injected saline (50 µL, 100 µg/mL); (2) Light, the mice were injected saline (50 µL, 100 µg/mL) and tumor was irradiated with 660 nm red light (30 mW/cm^2^, 5 min); (3) Pce6, the mice were injected PCe6 (50 µL, 100 µg/mL); (4) PCe6+Light, the mice were injected saline (50 µL, 100 µg/mL) and tumor was irradiated with 660 nm red light (30 mW/cm^2^, 5 min); (5) PCe6AZOM, the mice were injected PCe6AZOM (50 µL, 100 µg/mL); (6) PCe6AZOM+Light, the mice were injected PCe6AZOM (50 µL, 100 µg/mL) and tumor was irradiated with 660 nm red light (30 mW/cm^2^, 5 min).

**6.2 Hemolytic test**

Different concentrations of PCe6AZOM (50 µL, 100 µg/mL) were incubated with 2% red blood cells (RBCs) at 37 °C for 4 h and then cooled using an ice bath to stop hemolysis. After centrifugation at 1500 rpm for 15 min at 4 ℃, the supernatant was analyzed for hemoglobin at 576 nm using microplate reader. The percent of hemolysis was determined according to the following formula:

Hemolysis (%) = ($\frac{Sample absorbance - Background absorbance}{Positive control - Negative control}$) × 100%

The absorbance of the sample incubated with PCe6AZOM (50 µL, 100 µg/mL) in PBS was defined as background absorbance. The sample was incubated with PBS or deionized distilled water was defined as positive control and negative control, respectively.

**6.3 Animals and tumor model**

All protocols for animal studies conformed to the Guide for the Care and Use of Laboratory Animals and approved by the Dalian University of Technology Animal Care and Use Committee (DUT2020-028). The female BALB/c mice (5-6 weeks of age) were purchased from Liaoning Changsheng Biotechnology Co., Ltd. The breast cancer mouse model (4T1 tumor-bearing mice) were established through injecting into the armpit positions with 5×10^6^ 4T1 cells subcutaneous. The 4T1 tumor-bearing mice were used for fluorescence imaging and PDT, when the tumors reached about 100 mm^3^ in volume.

**6.4 For imaging *in vivo***

To study the *in vivo* tumor accumulation and retain, free Ce6 (50 µL, 100 µg/mL) and PCe6AZOM (50 µL, 100 µg/mL) were intravenously injected into mice, respectively. The mice anaesthetization with 2% isoflurane in oxygen, and fluorescence imaging *in vivo* was observed by using the small animals imaging system at different time after injection. For the biodistribution of free Ce6 and PCe6AZOM further study *in vivo*, 4T1 tumor bearing Balb/c mice were euthanized after intravenously injection 72 h. Major organs (including tumor, heart, liver, spleen, lung, kidney) from mice were collected and imaged by the small animals imaging system. Then the major organs evaluated fluorescence intensity by using Spectrum Living Image 4.0. The excitation wavelength was 640 nm, and the collected emission wavelength was 660-750 nm.

**6.5 *In vivo* anti-tumour experiment**

To investigate the anti-tumor efficacy *in vivo*, all 4T1 tumor-bearing mice were randomly divided into six groups (n = 5), and different reagents were intravenously injected into mice and different treatments were conducted including the following groups: (1) Control, the mice were injected saline (200 µL, 100 µg/mL); (2) Light, the mice were injected saline (50 µL, 100 µg/mL) and tumor was irradiated with 660 nm red light at a power density of 100 mW/cm^2^ for 5 min after post-injection 48 h; (3) Pce6, the mice were injected PCe6 (50 µL, 100 µg/mL); (4) Pce6+Light, the mice were injected saline (50 µL, 100 µg/mL) and tumor was irradiated with 660 nm red light at a power density of 100 mW/cm^2^ for 5 min after post-injection 48 h; (5) Pce6AZOM, the mice were injected PCe6AZOM (50 µL, 100 µg/mL); (6) Pce6AZOM+Light, the mice were injected PCe6AZOM (50 µL, 100 µg/mL) and tumor was irradiated with 660 nm red light at a power density of 100 mW/cm^2^ for 5 min after post-injection 48 h.

The body weight of the mice were measured and the tumor volume were recorded every other day for a span of 14 days. The tumor volumes were calculated by the following equation: width × width × length/2. The mice were euthanized in the last treatment-day and and tumor tissues of mice were collected for weighing. The main organs (including heart, liver, spleen, lung, kidney) and tumors of the mice were isolated and fixed in 10% formalin for staining with hematoxylin-eosin (H&E).

**7. Statistical analysis**

Calculated data are expressed as mean values±SD. Statistical comparisons between groups were performed using the Two-tailed Student’s t-test. All statistical analysis was performed with Origin 2019b software and Graphpad Prism 8.0 software. The statistical significance differences was defined as *P < 0.05, ** P < 0.01 and *** P < 0.001.


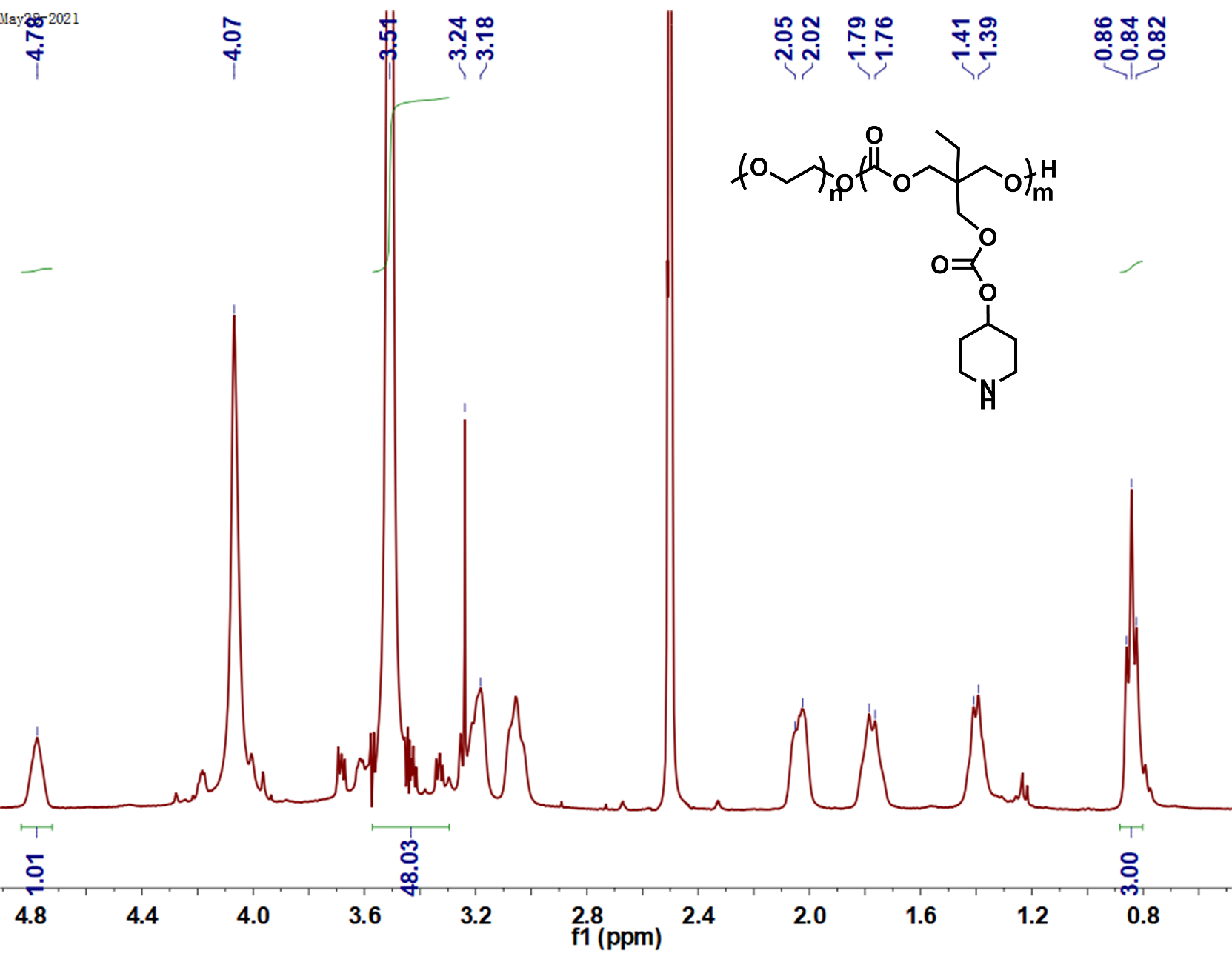


**Figure S3.** ^1^H NMR spectrum of **MPEG-b-PTMCP** (400 MHz, DMSO-d_6_).


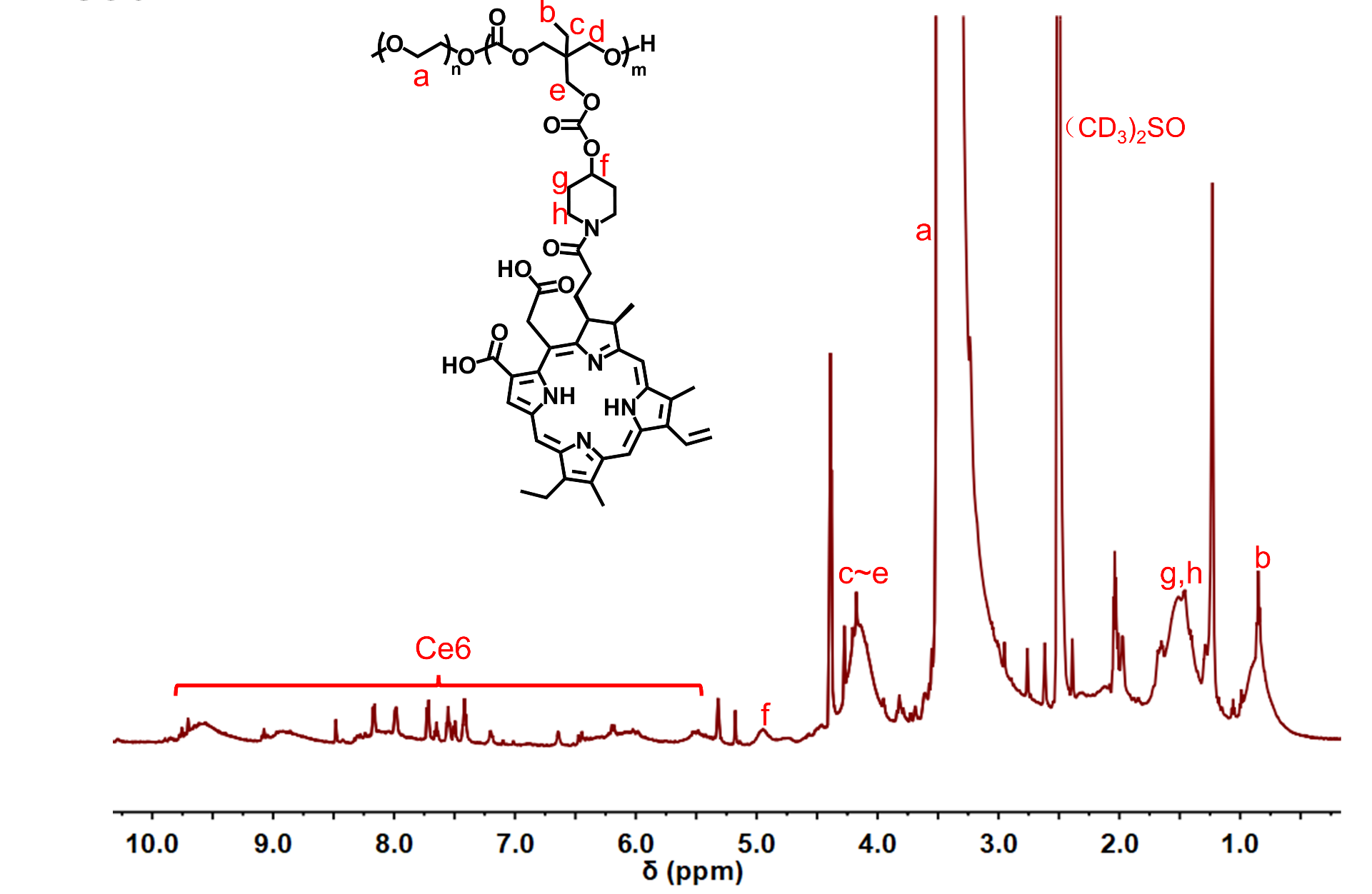


**Figure S4.** ^1^H NMR spectrum of **PCe6** (400 MHz, DMSO-d_6_).


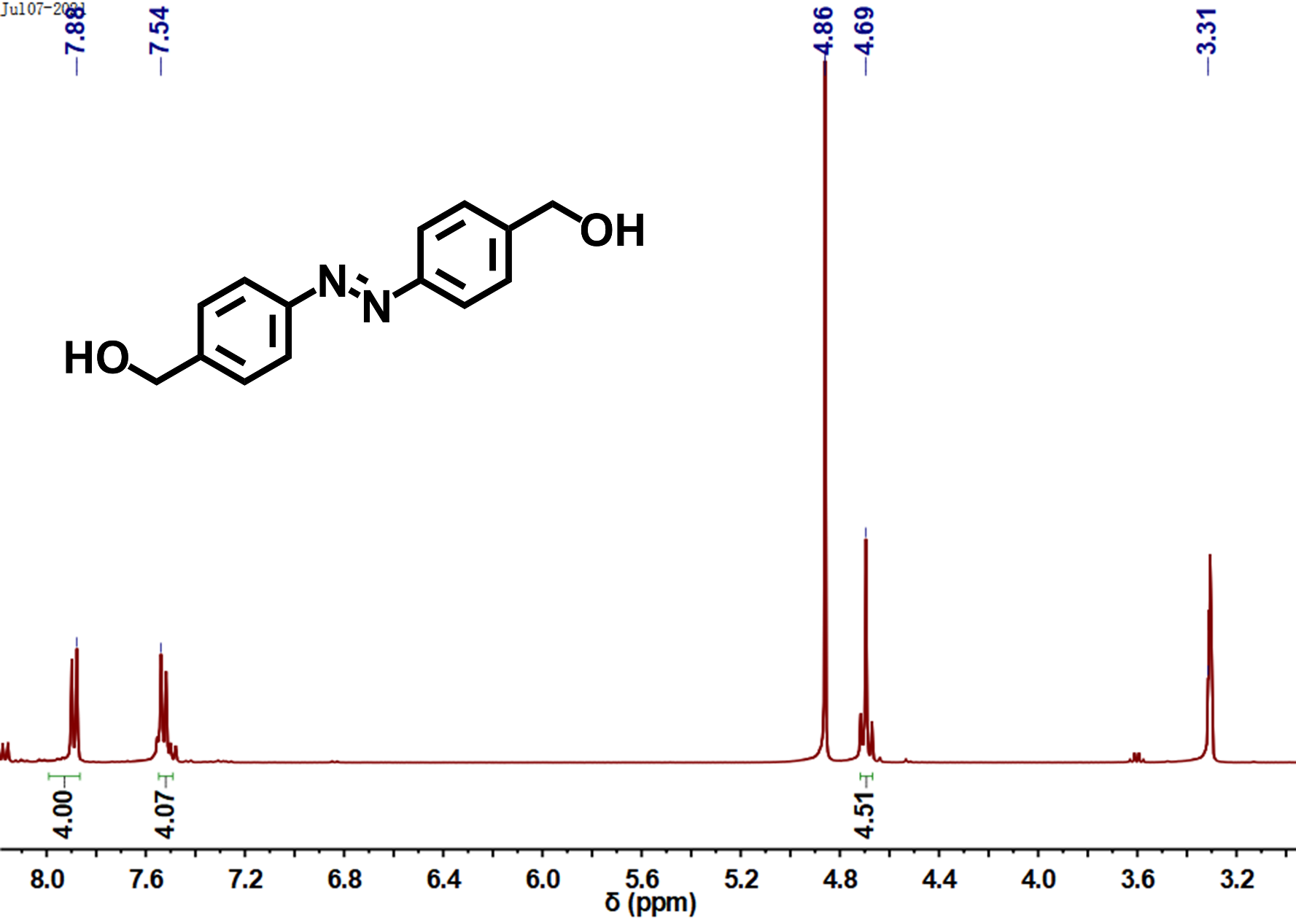


**Figure S5.** ^1^H NMR spectrum of **Compound 1** (400 MHz, CD_3_OD).


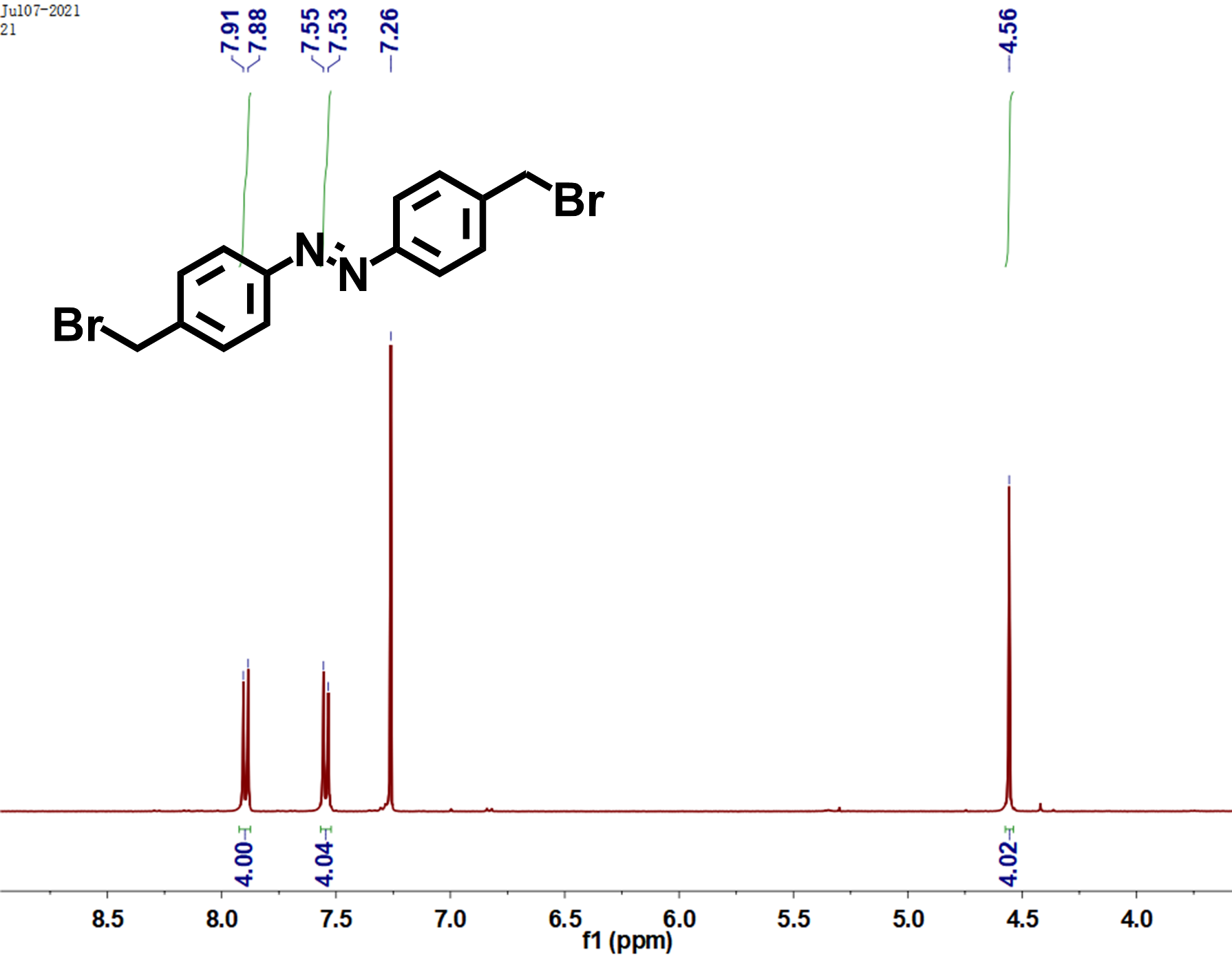


**Figure S6.** ^1^H NMR spectrum of **Compound 2** (400 MHz, CDCl_3_).


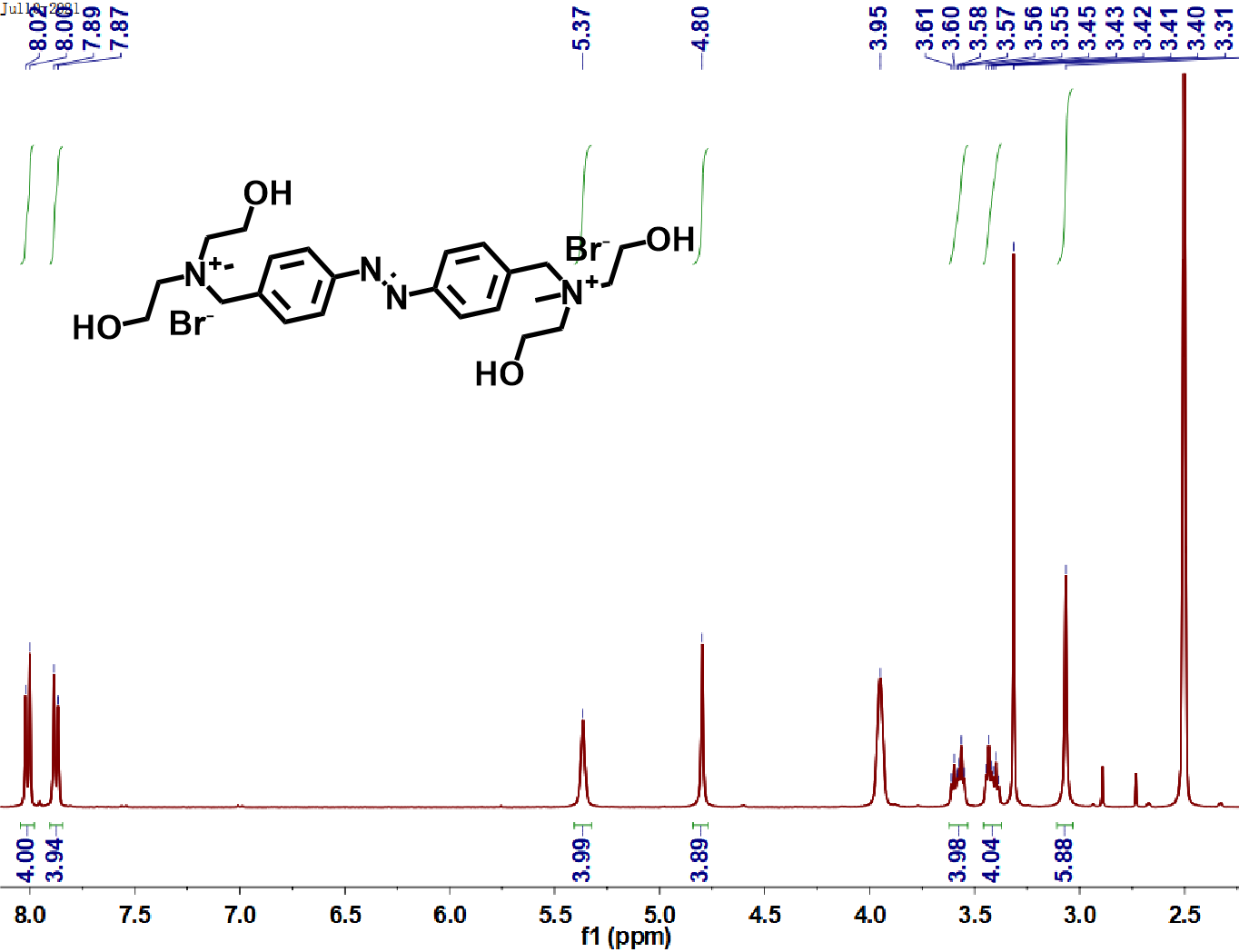


**Figure S7.** ^1^H NMR spectrum of **Compound 3** (400 MHz, DMSO-d_6_).


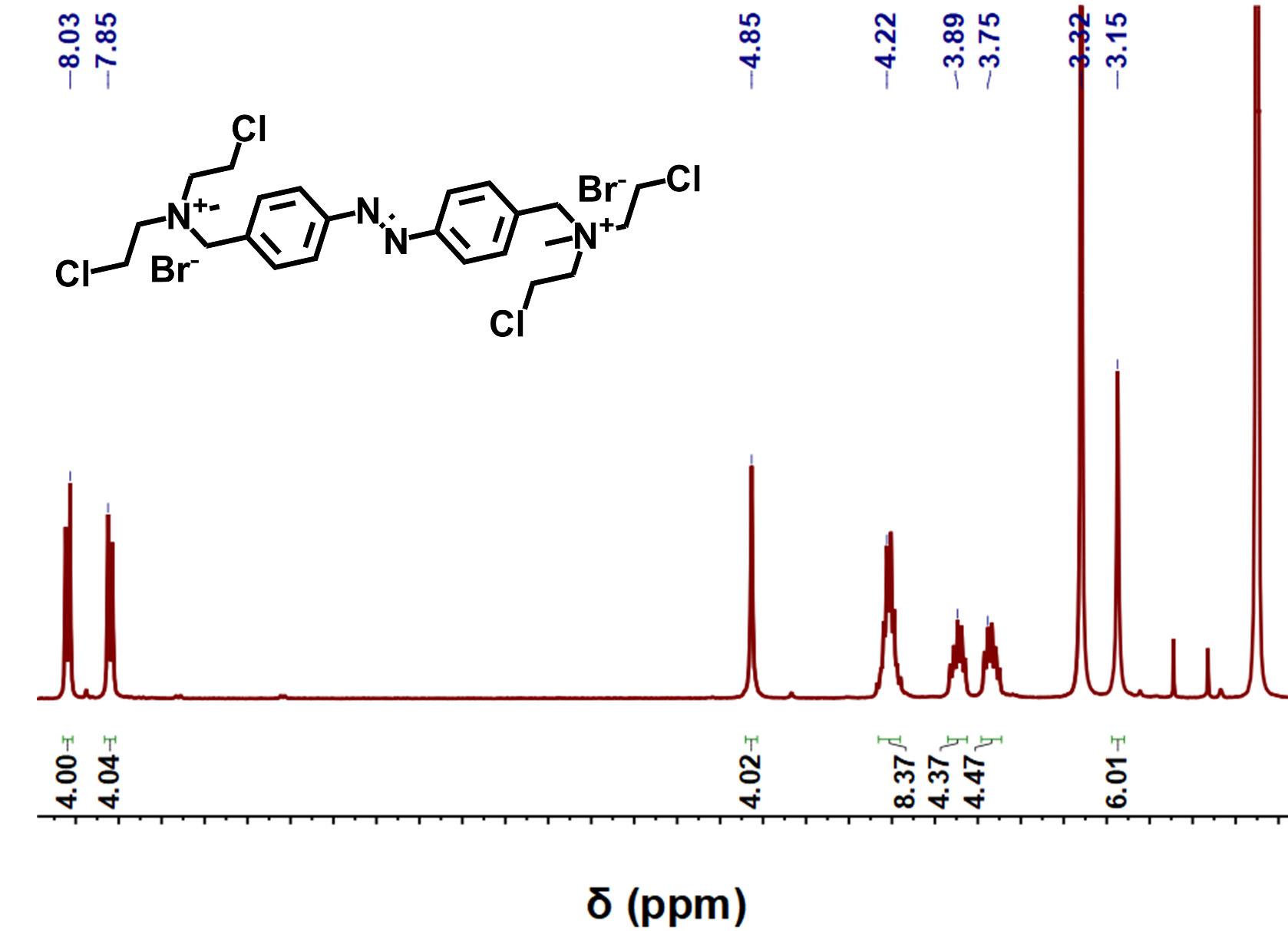


**Figure S8.** ^1^H NMR spectrum of **Compound 4** (400 MHz, DMSO-d_6_).


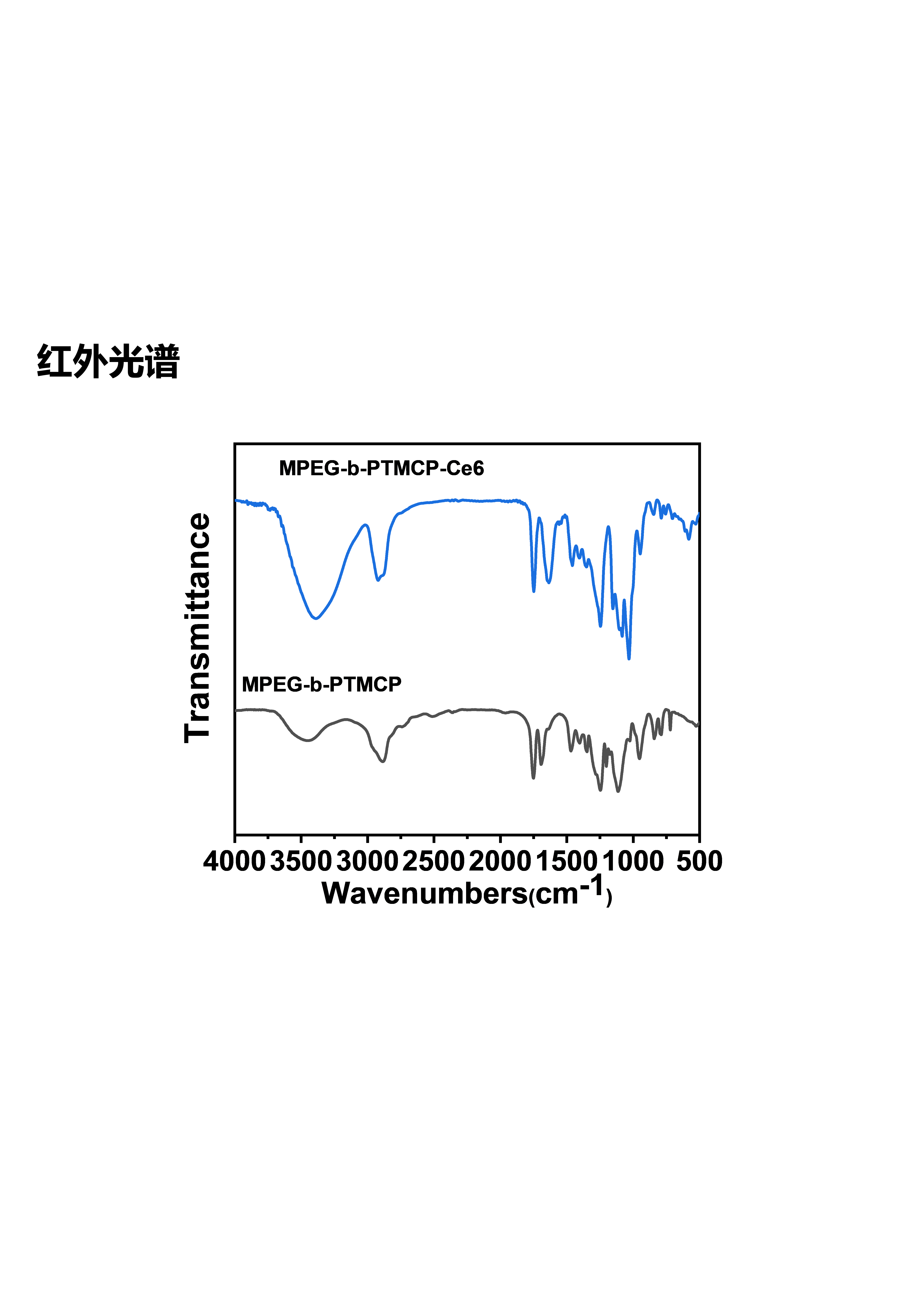


**Figure S9.** The infrared absorption spectrum of PCe6 and MPEG-b-PTMCP.


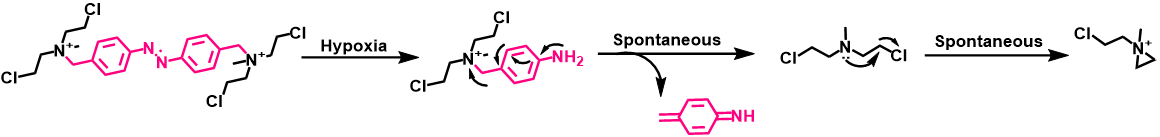


**Figure S10.** The mechanism of pro-drug AZOM action in hypoxia.


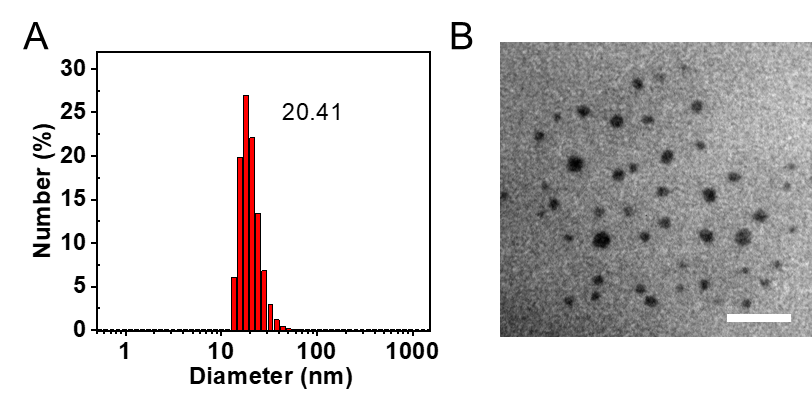


**Figure S11.** a) DLS size distribution data of PCe6 nanoparticles. b) TEM images of PCe6nanoparticles. Scale bar: 20 nm.


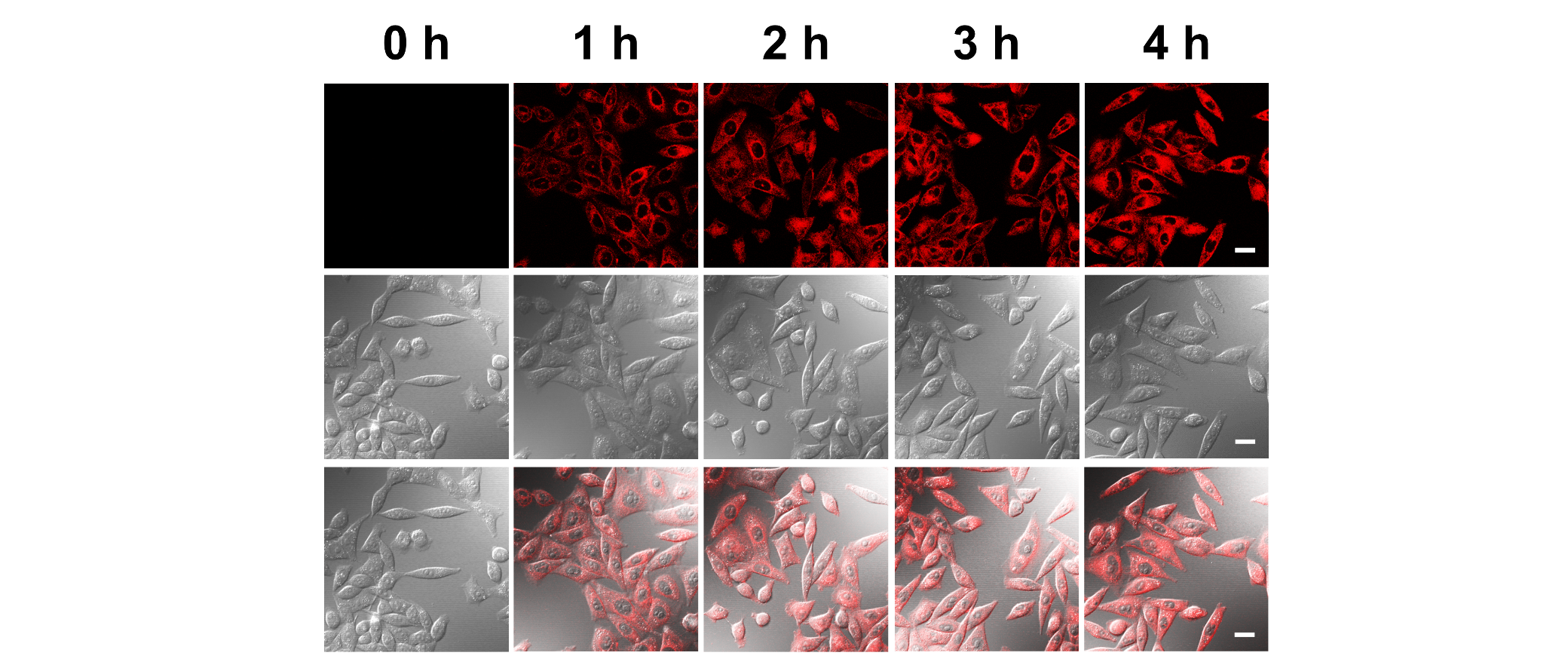


**Figure S12.** CLSM images of Pe6AZOM uptake in MCF-7 cells. Scale bar: 30 µm.


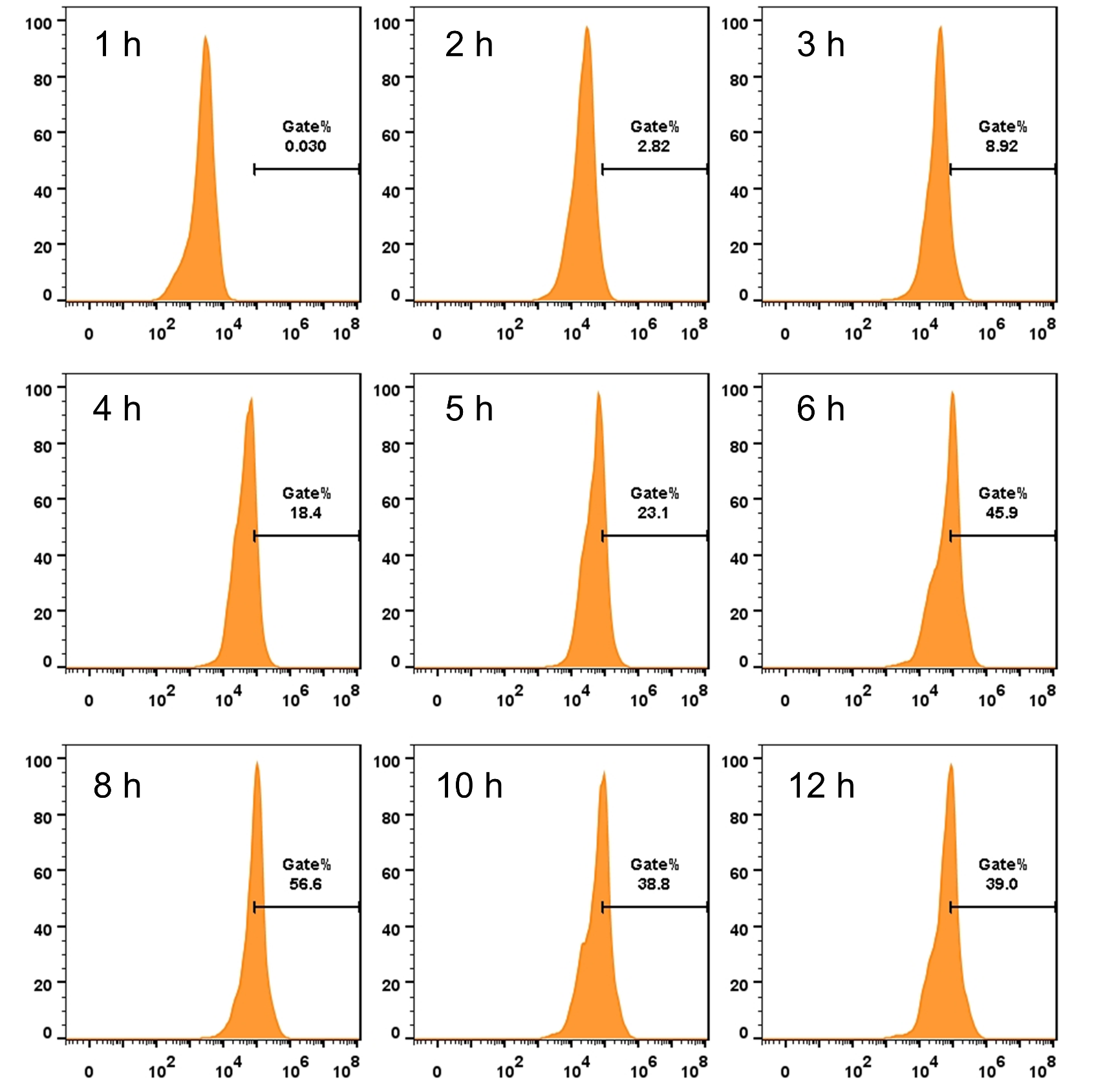


**Figure S13.** Flow cytometric quantitative fluorescence intensities analysis of MCF-7 cells intracellular PCe6AZOM after treatment with different time.


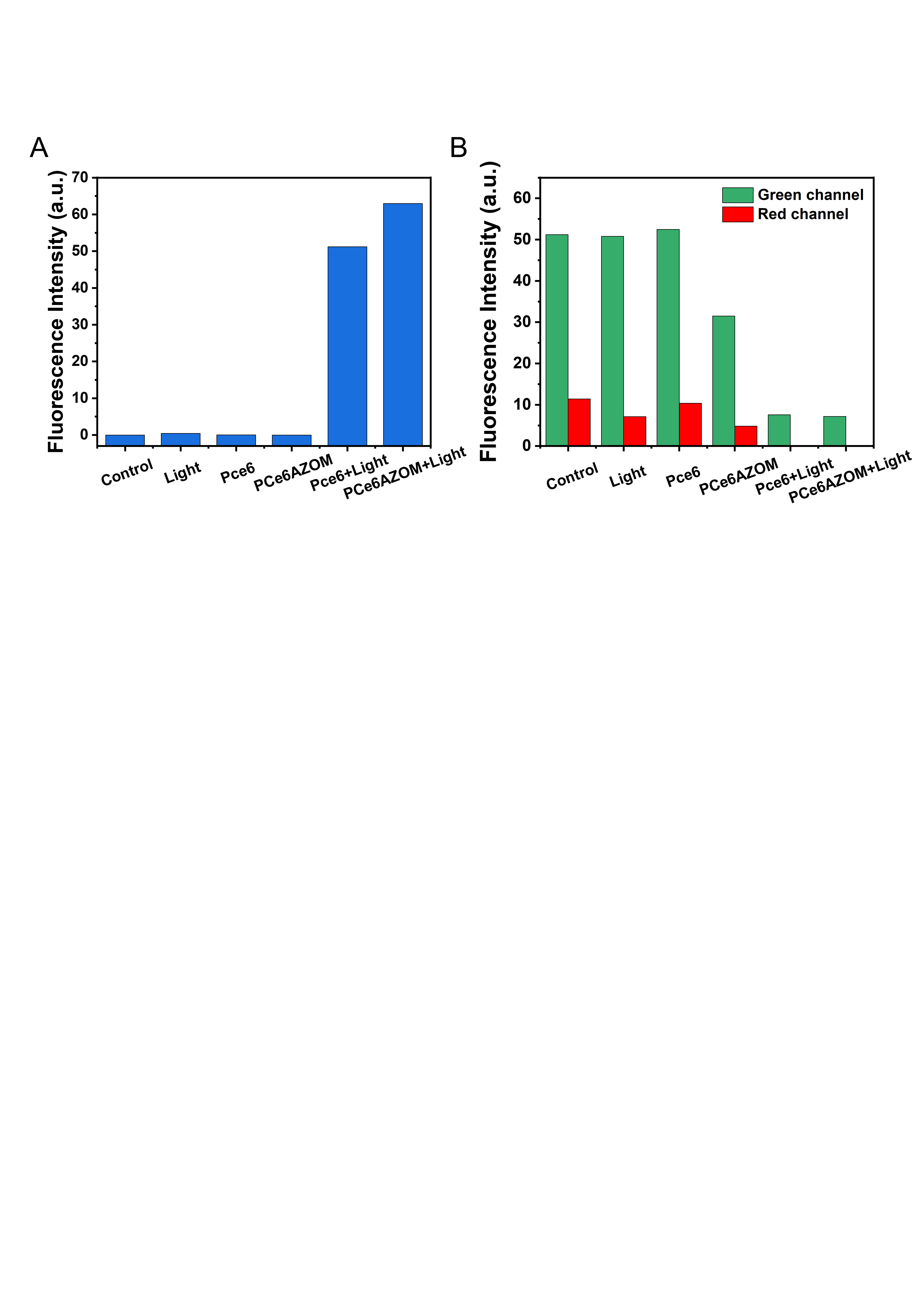


**Figure S14.** A) Quantitative analysis of DCFH-DA fluorescence intensity. B) Quantitative analysis of AO fluorescence intensity in green and red channel.


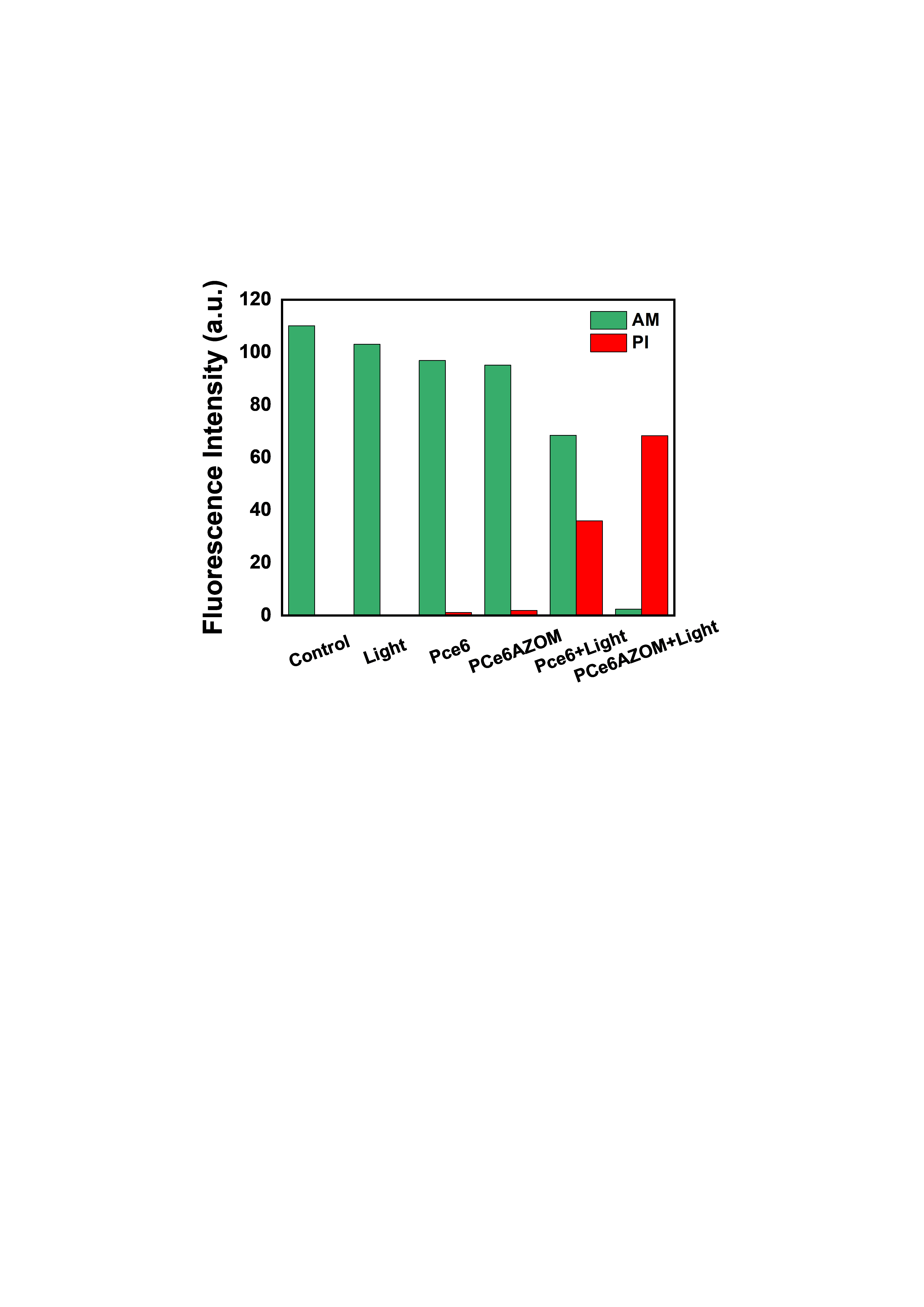


**Figure S15.** Quantitative analysis of AM and PI fluorescence intensity.


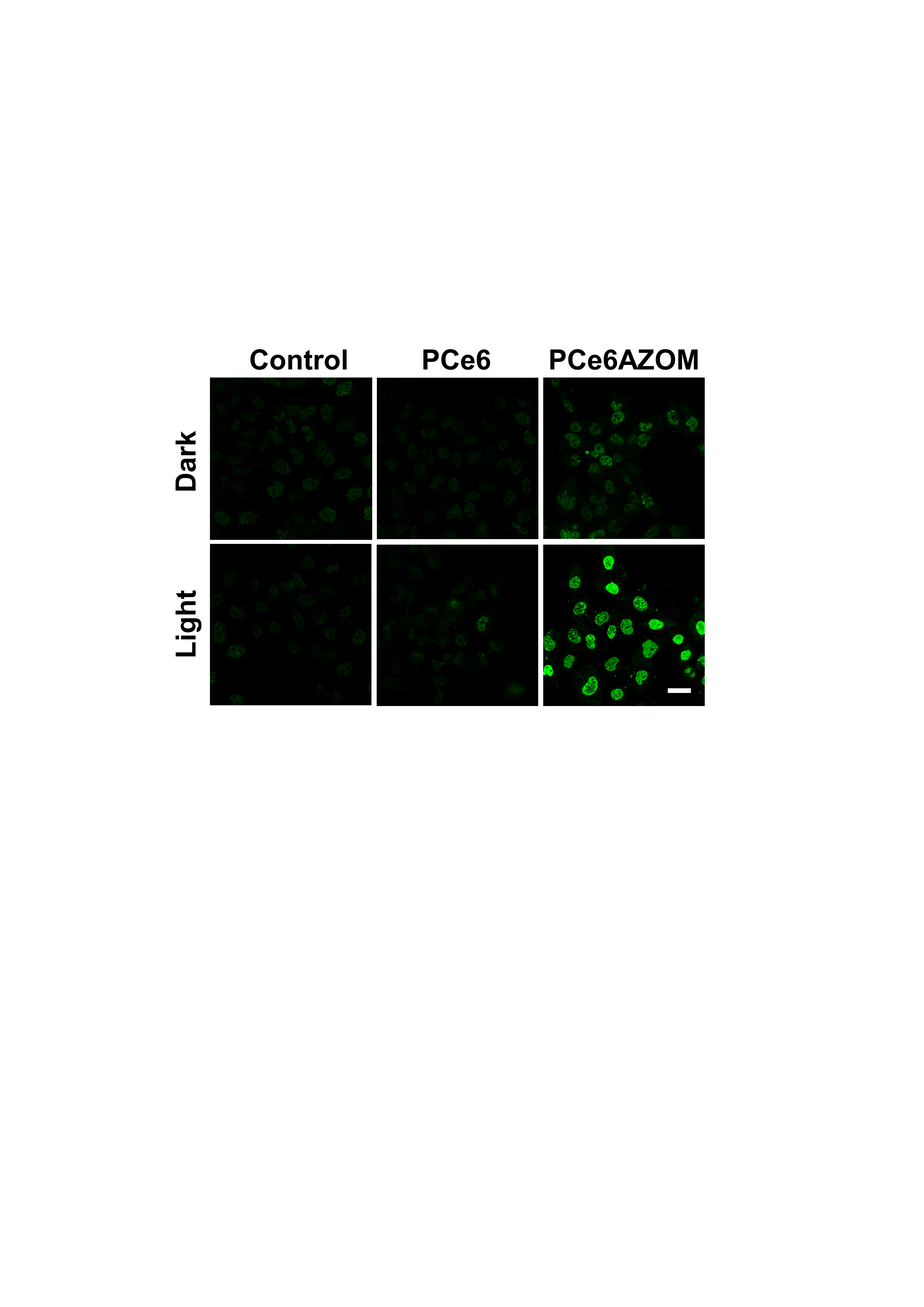


**Figure S16.** Confocal laser scanning microscopy images of γ-H2AX (a DNA damage marker) in MCF-7 cells after various treatments. Scale bar: 30 µm.


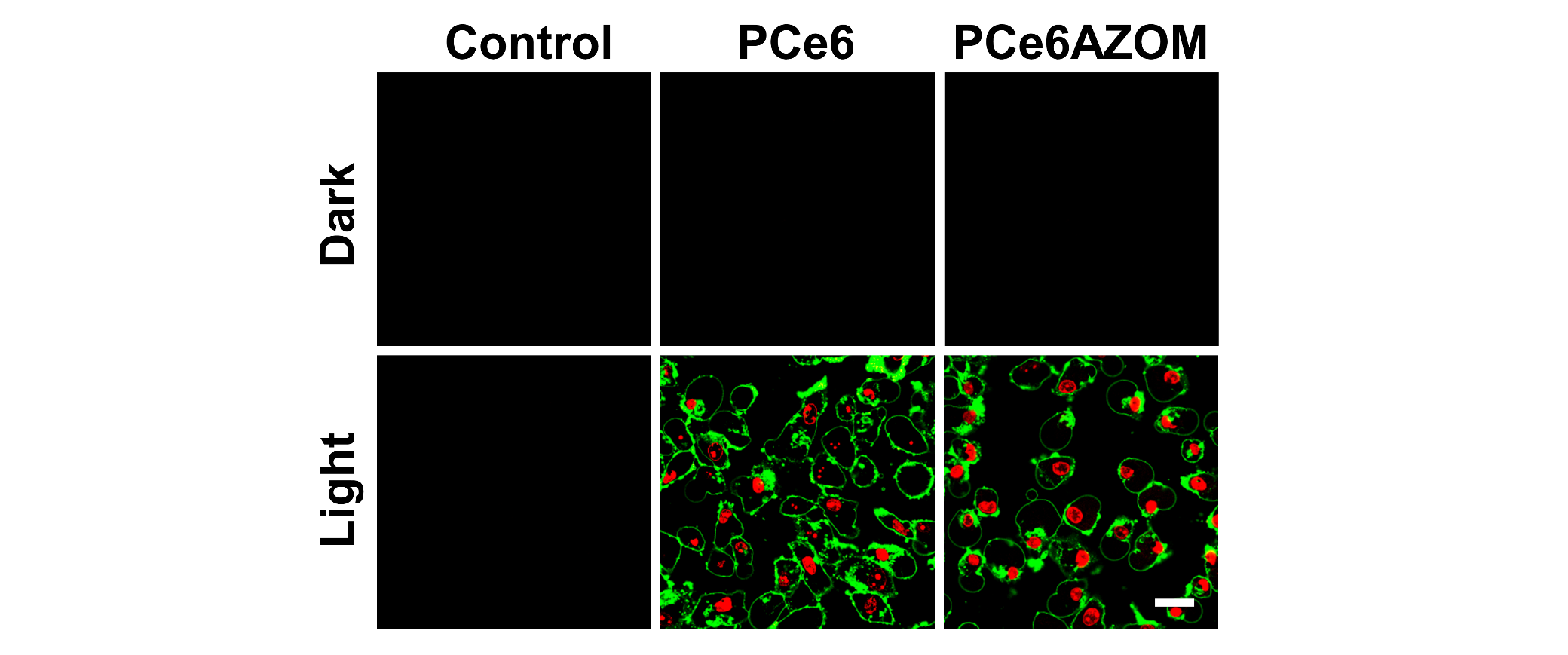


**Figure S17.** Cell apoptosis images of MCF-7 cells with different treatment stained with Annexin V-FITC/PI by CLSM. Scale bar: 30 µm.


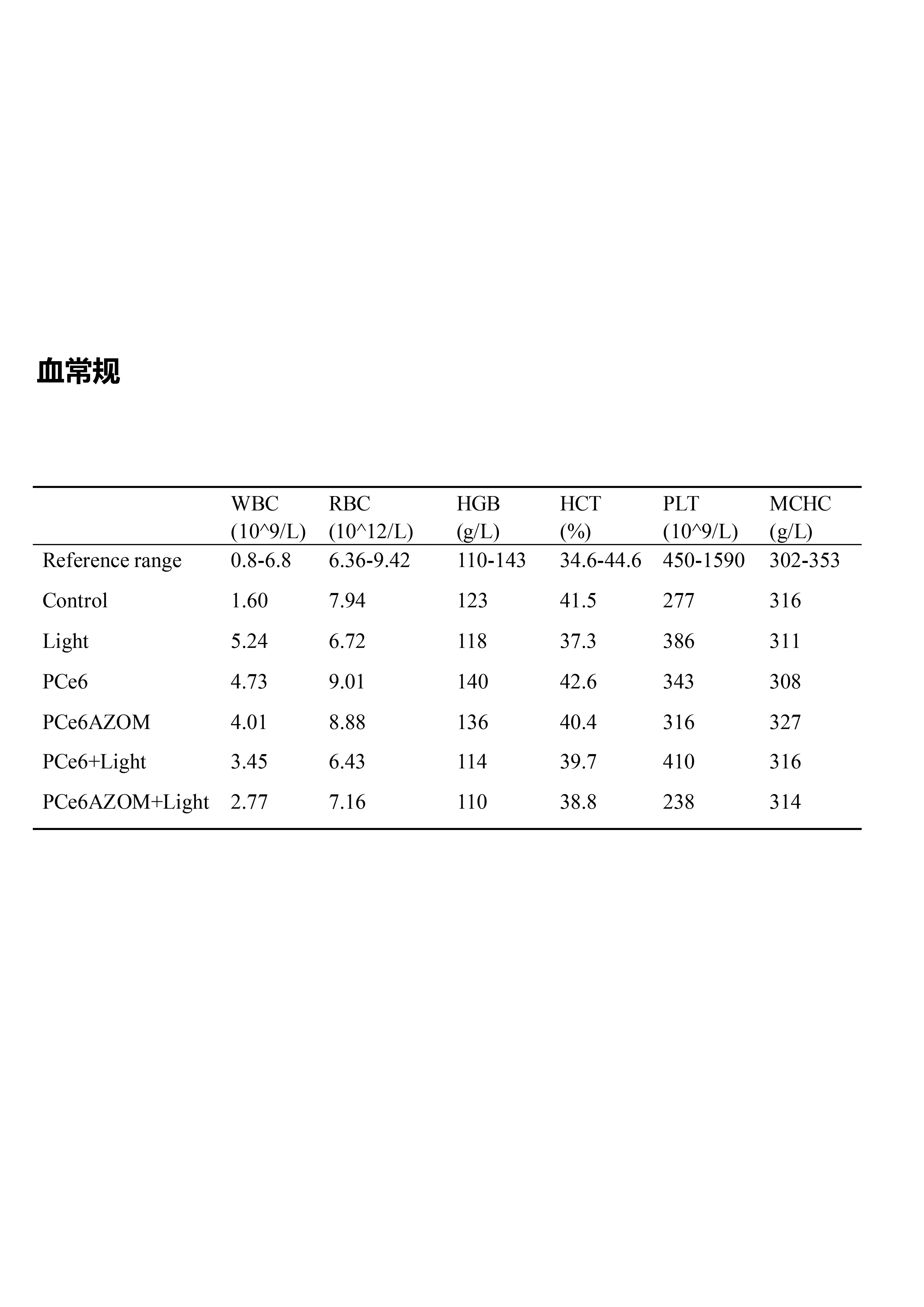


**Figure S18.** Blood biochemistry analysis of mice after different treatment after 14 days. The tested parameters including white blood cells (WBC), red blood cells (RBC), hemoglobin (HGB), hematocrit (HCT), platelets (PLT), and mean corpuscular hemoglobin concentration (MCHC). The obtained data were based on 3 mice.


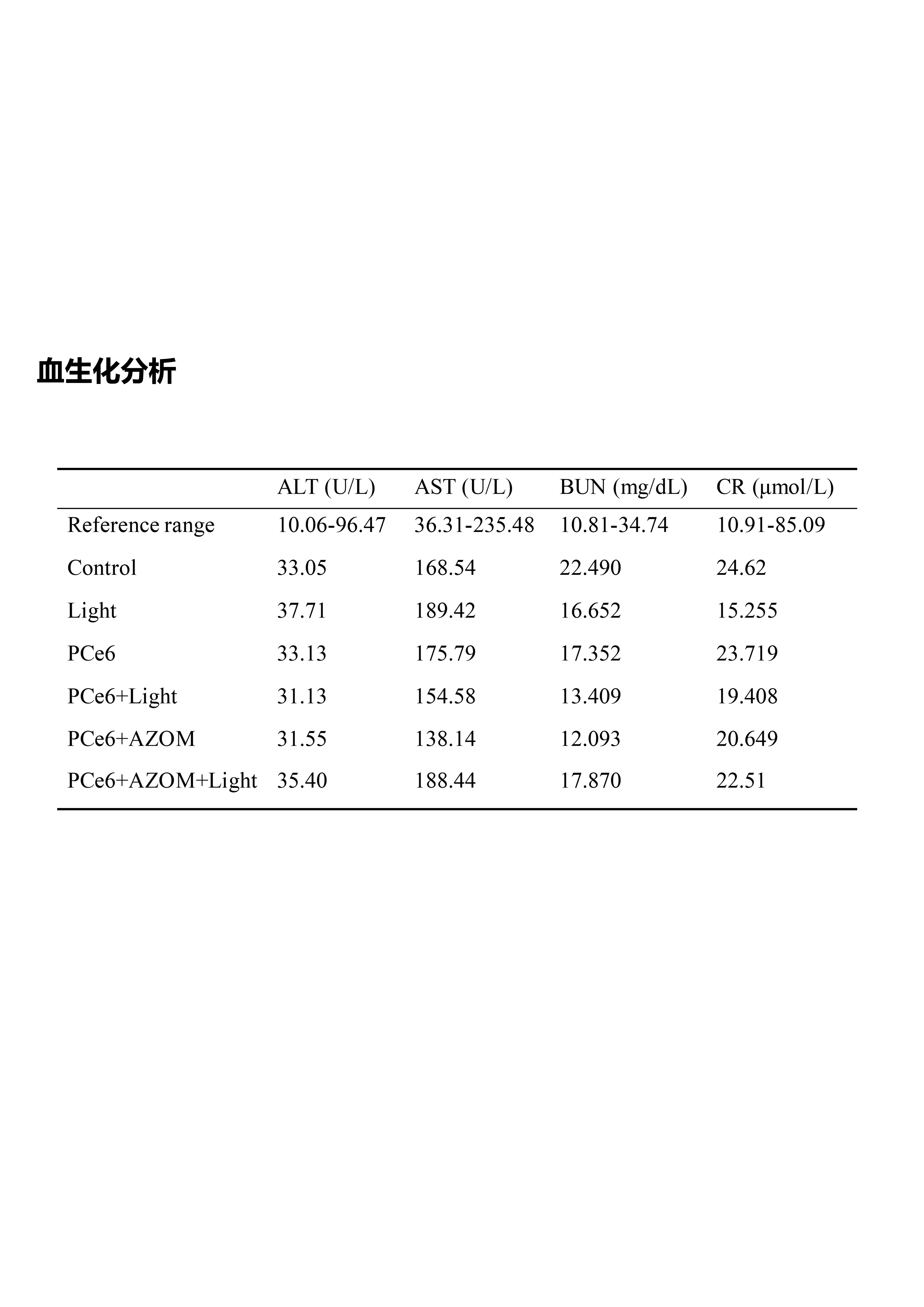


**Figure S19** The routine blood analysis of mice after different treatment after 14 days. The tested parameters including alanine aminotransferase (ALT), aspartate aminotransferase (AST), blood urea nitrogen (BUN), creatinine (CR). The obtained data were based on 3 mice.


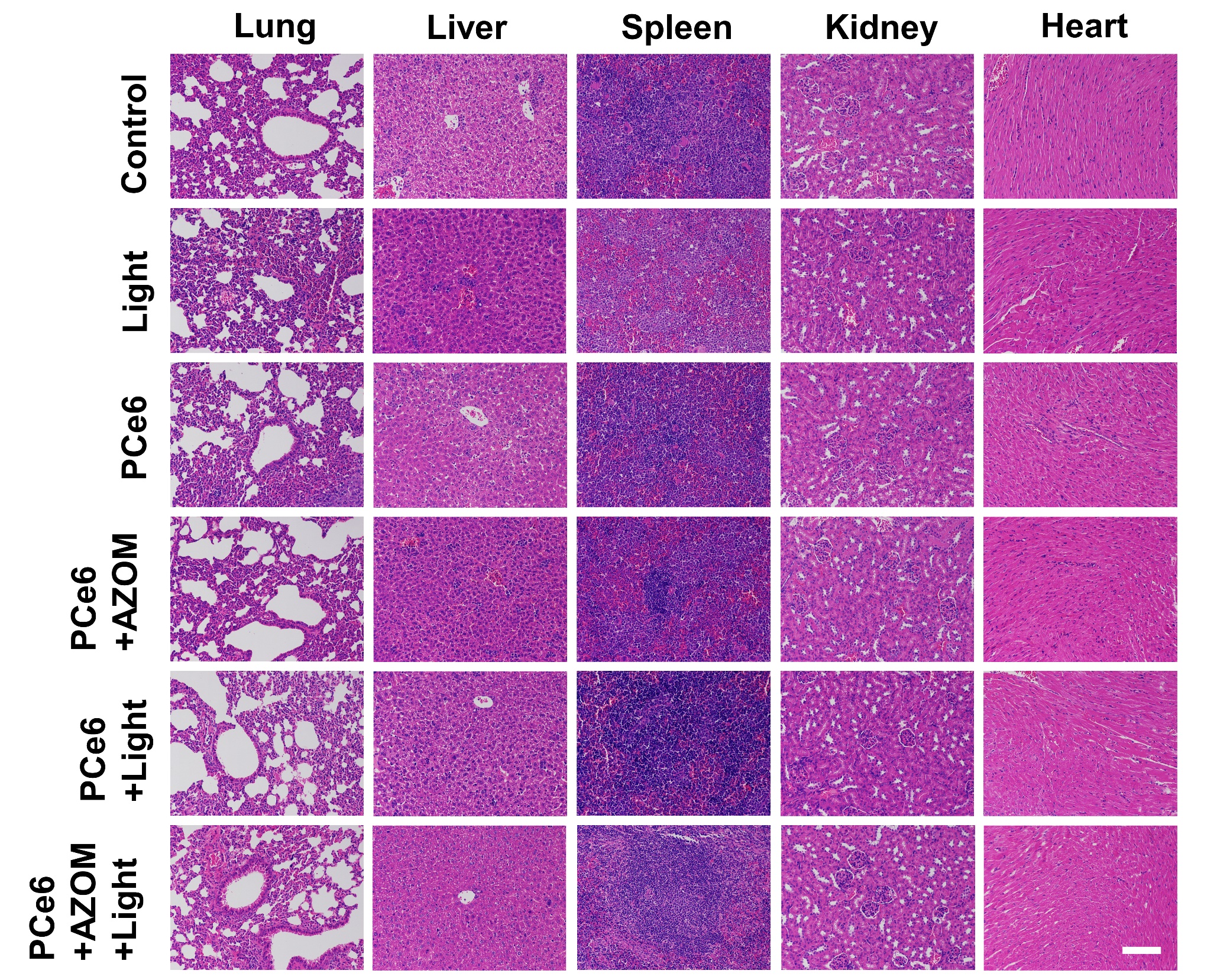


**Figure S20.** Histological hematoxylin and eosin (H&E) analysis of the mice including heart, liver, spleen, lung, and kidney tissues collected in the different groups at the 14 days treatment. Scale bar: 100 μm.
